# Nanocomposite formulation for a sustained release of free drug and drug-loaded responsive nanoparticles: an approach for a local therapy of glioblastoma multiforme

**DOI:** 10.1038/s41598-023-32257-5

**Published:** 2023-03-29

**Authors:** Luiza C. S. Erthal, Yang Shi, Kieron J. Sweeney, Oliviero L. Gobbo, Eduardo Ruiz-Hernandez

**Affiliations:** 1 School of Pharmacy and Pharmaceutical Sciences and Trinity St. James’s Cancer Institute, Panoz Institute, Trinity College Dublin, College Green, Dublin 2, Ireland; 2 Department of Nanomedicine and Theranostics, Institute for Experimental Molecular Imaging, RWTH Aachen University Clinic, Forckenbeckstrasse 55, 52074 Aachen, Germany; 3 National Neurosurgical Centre, Beaumont Hospital, Dublin 9, Ireland; 4 Royal College of Surgeons in Ireland, Dublin 2, Ireland

**Keywords:** Materials science, Nanoscience and technology

## Abstract

Malignant gliomas are a type of primary brain tumour that originates in glial cells. Among them, glioblastoma multiforme (GBM) is the most common and the most aggressive brain tumour in adults, classified as grade IV by the World Health Organization. The standard care for GBM, known as the Stupp protocol includes surgical resection followed by oral chemotherapy with temozolomide (TMZ). This treatment option provides a median survival prognosis of only 16–18 months to patients mainly due to tumour recurrence. Therefore, enhanced treatment options are urgently needed for this disease. Here we show the development, characterization, and in vitro and in vivo evaluation of a new composite material for local therapy of GBM post-surgery. We developed responsive nanoparticles that were loaded with paclitaxel (PTX), and that showed penetration in 3D spheroids and cell internalization. These nanoparticles were found to be cytotoxic in 2D (U-87 cells) and 3D (U-87 spheroids) models of GBM. The incorporation of these nanoparticles into a hydrogel facilitates their sustained release in time. Moreover, the formulation of this hydrogel containing PTX-loaded responsive nanoparticles and free TMZ was able to delay tumour recurrence in vivo after resection surgery. Therefore, our formulation represents a promising approach to develop combined local therapies against GBM using injectable hydrogels containing nanoparticles.

## Introduction

For more than 20 years, the standard of care for patients diagnosed with glioblastoma multiforme (GBM) has remained unchanged. When the location and size of the tumour permit it, surgical resection is performed. Following this, the patient receives radiotherapy and oral chemotherapy with temozolomide (TMZ)^1^. This chemotherapeutic drug is an alkylating agent that acts by inducing base mismatches and DNA breaks, which impair cell replication^2^. Thus, this drug is very active during oncogenesis, and radiotherapy plays a synergistic effect damaging the DNA of cancer cells, leading them to death^3^. Although TMZ can cross the blood–brain barrier to exert its effect, the high dose required for systemic treatment could induce serious side effects^4^. Moreover, this treatment option provides a median survival prognosis of only 16–18 months to patients, mainly due to tumour recurrence.

New formulations are focusing on the localized treatment of GBM, so that effective drug concentrations can be achieved on the tumour site avoiding the severe side effects of systemic delivery. Some strategies are already in place to deliver drugs directly into the brain such as convection-enhanced delivery^5,6^, intranasal delivery^7,8^ and, specifically for GBM, the implantation of carmustine wafers, known commercially as Gliadel^9^, after tumour resection surgery.

Although the use of Gliadel shows improvement in the treatment of some patients, it also presents some challenges related to the amount of drug that can reach the tumour cells^10,11^ and post-surgical complications after implantation^12,13^. For these reasons, the research and development of more effective local delivery approaches to the brain are crucial to improving GBM treatment outcomes post-surgery.

Injectable hydrogels are being explored in biomedical applications for the local delivery of drugs^14^. Moreover, composite materials that explore the advantages of hydrogel with nanotechnology deserve special attention. Some composite materials have shown very promising effects against GBM in preclinical models by increasing significantly the median survival time of the animals when compared to other treatments such as free drugs or drug-loaded nanoparticles alone^15–17^. Additionally, a common argument from these studies is that the nanocomposite formulations can ensure the sustained and prolonged release of drugs toward tumour cells, which improves treatment efficiency. Nanoparticles may increase the concentration of drugs in the tumour site and keep them for a longer time. Specifically, mesoporous silica nanoparticles (MSN) can be designed to increase the penetration in certain tissues^18^, to be hidden from the immune system^19^ and also to be responsive to different types of stimuli such as environmental pH^20,21^ and redox conditions^22,23^. Besides their responsiveness, the nanoparticle surface functionalization permits the strategical pore closure to control the release and target the therapy to specific sites^24^.

**Figure 1:**
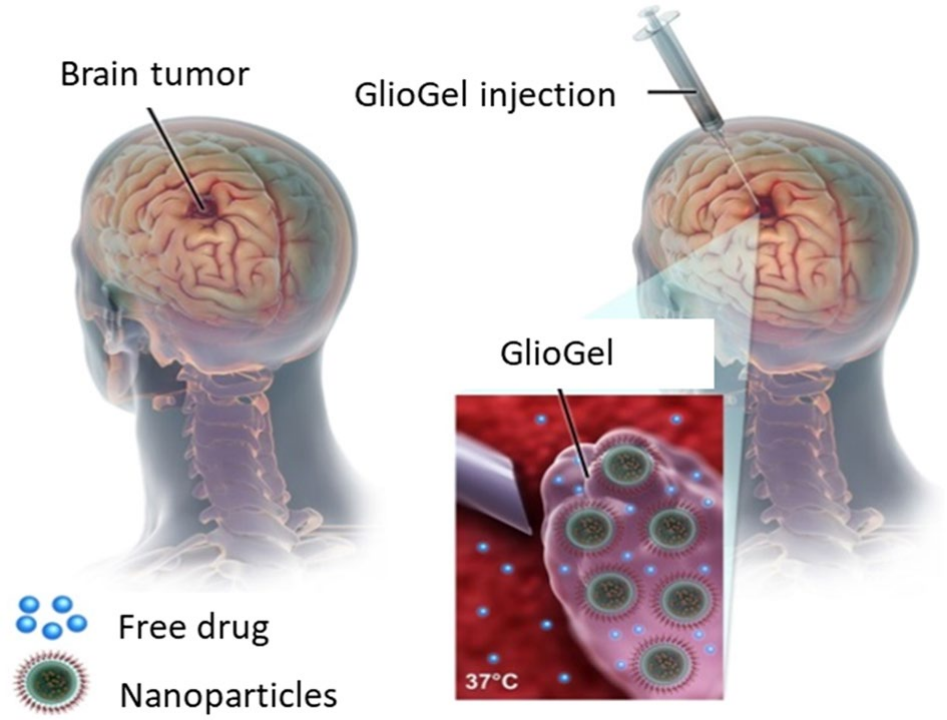
GlioGel formulation, composed of free temozolomide and stimuli-responsive paclitaxel-loaded MSN, can be injected in the tumour cavity after resection surgery.

In this study, we developed, characterized and evaluated a new formulation, named GlioGel (Fig. 1), which is composed of an injectable hydrogel loaded with free temozolomide and stimuli-responsive paclitaxel-loaded MSN to treat GBM locally post-surgery. Firstly, we designed and characterized responsive MSN loaded with paclitaxel and analysed their effect against a human glioblastoma cell line. We incorporated a redox-responsive molecular gate^25^, using polyethylene glycol (PEG) attached through a disulphide bond on the surface of the nanoparticles, to allow a preferential release of the chemotherapeutic drug from the mesoporous matrix in the intracellular environment. Secondly, we investigated the ability of the combination of free temozolomide and paclitaxel-loaded MSN to inhibit spheroids growth in vitro compared to single treatments. Finally, we combined the free temozolomide and paclitaxel-loaded MSN into an injectable hydrogel and evaluated this novel therapeutic formulation in a mouse model of GBM post-surgery.

## Materials and methods

### Materials

Fetal bovine serum (FBS), fluorescein isothiocyanate (FITC), glutathione (GSH), L-glutamine solution, Minimum Essential Medium Eagle (MEM), poly(ethylene glycol) (PEG) methyl ether thiol, sodium hydroxide (NaOH), temozolomide (TMZ), paclitaxel (PTX), tetraethyl orthosilicate (TEOS), trypsin–EDTA solution 0.25%, 2,2-dipyridyl disulphide (Aldithriol-2), (3-mercaptopropyl) trimethoxysilane, (3-aminopropyl)triethoxysilane (APTES), sodium acetate, Triton X-100, Tween 80, acetonitrile (ACN), dimethyl sulfoxide (DMSO) and p-nitrophenyl phosphate were purchased from Sigma- Aldrich Ireland. Cetyltrimethylammonium bromide (CTABr) was purchased from ThermoFisher Scientific, Ireland. Cell viability CCK-8 kit was purchased from Dojindo, Europe. Ultra-pure distilled water was prepared by the PURELAB Option system (ELGA LabWater, UK). Poly(1-(acetonylamino)-2-methyl-2-propen-1-one) was synthesized according to a previously published method^26^. The molecular weight of the obtained polymer was determined by ^1^H NMR and GPC. The number-average molecular weight of the polymer was 8.5 kDa and the polydispersity index was 1.1.

### Methods

#### Mesoporous silica nanoparticles synthesis

The synthesis of the mesoporous silica nanoparticles (MSN) was performed following the “liquid crystal templating” method^27^. Briefly, cetyltrimethylammonium bromide (1 g) was added to distilled water (500 mL) at 30 °C. When it was perfectly dissolved, NaOH 2 M (3.5 mL) was added and the solution was heated until 80 °C. At this temperature, the stirring rate was increased and 5 mL of tetraethyl orthosilicate was added. This mixture was stirred for 2 h until full hydrolysis and condensation of the silica precursor. The resulting nanoparticles were kept on the bench to cool down until room temperature. Finally, the nanoparticle dispersion was centrifuged at 5000 rpm for 20 min to isolate the MSN, washed twice with distilled water and once with ethanol, where it was kept until surfactant extraction.

To synthesize fluorescently labelled nanoparticles (MSN-FITC), fluorescein isothiocyanate (FITC) was mixed with 3-aminopropyltriethoxysilane (APTES) for 2 h in the dark. Then, tetraethyl orthosilicate was added^28^.

An acidic extraction was performed to remove the CTABr surfactant from the material. The nanoparticles (1 g) were re-suspended in 1 M HCl in ethanol (100 mL) and put in a reflux system under agitation and heating to 80 °C overnight. After that, the material was washed with distilled water four times and once with ethanol, where it was kept until further use^29^.

#### Mesoporous silica nanoparticles loading and functionalization

To load the nanoparticles with a model dye, a ratio of 0.8 mmol of Safranin-O per gram of nanoparticles was used. The mixture was stirred in water overnight at room temperature^25^.

To load the nanoparticles with PTX, the drug (2.5 mg/ml) was dissolved in dichloromethane (10 ml) and this solution was added to the nanoparticles (75 mg)^30^. This mixture was kept stirring overnight at room temperature.

Before proceeding with PEG functionalization, the loaded nanoparticles were collected by centrifugation and resuspended in ACN in the presence of an excess of Safranin-O or PTX, and 3 mmol of (3-mercaptopropyl) trimethoxy-silane was added. This mixture was stirred for 5.5 h at room temperature. After this period, 3 mmol of 2,2ʹ-dipyridyl disulphide (Aldrithiol) was added to the reaction mixture and kept stirring overnight at room temperature. Then, the sample was centrifuged and dried under vacuum. The dried solid and poly(ethylene glycol) methyl ether thiol were resuspended in ACN in the presence of an excess of Safranin-O or PTX, and the mixture was again stirred overnight. The final materials, Safranin-O-loaded MSN-PEG and PTX-loaded MSN-PEG were isolated by centrifugation, washed and dried under vacuum^25^.

#### Drug loading estimation by high-performance liquid chromatography

The encapsulation efficiency (EE%) and loading capacity (LC%) were estimated using High-Performance Liquid Chromatography (HPLC) with UV–visible detection of PTX before and after the loading procedure, and were calculated as follows:

$$EE\%=\frac{(total \,drug \,added-free \,non-loaded\, drug)}{total \,drug\, added}$$

$$LC\%=\frac{amount\,of \,total \,drug\, loaded}{total\, nanoparticle\, weight}$$

Drug quantification was performed by HPLC using a Gemini 5 μm C18 column (110 Å, 250 × 4.6 mm) attached to a Waters Alliance HPLC System following a previously described method for paclitaxel^31^. A mobile phase consisting of 70% ACN/30% water (v/v) was used with a flow rate of 1 ml/min and the detection wavelength was 227 nm.

#### Hydrogel preparation

The hydrogel composition was selected due to its mechanism of covalent polymer self-assembly^32^. Poly(1-(acetonylamino)-2-methyl-2-propen-1-one)^26^ was dissolved in water at a concentration of 80 mg/ml. For crosslinking, 25 µl of the polymer solution was mixed with the same volume of adipic acid dihydrazide crosslinker solution in water (24.3 mg/ml). The mixture (50 µl) was left at room temperature until the solution was gelled.

### Materials characterization

#### Nanoparticle characterization

Mesoporous silica nanoparticles were characterized by standard methods briefly described below^29^.

The MSN were visualized by transmission and scanning electron microscopy (TEM and SEM) before and after surface functionalization. For TEM, an MSN suspension in ethanol was dropped into holey carbon-coated 200 mesh copper grids and subsequently left to dry at room temperature. Images were acquired in a JEOL 2100 microscope operating at 200 kV. The average size of the nanoparticles was measured with ImageJ software.

For SEM analysis, the MSN suspension was dropped in a metal stub and left to dry at room temperature. Samples were then coated with a thin layer of gold/palladium under vacuum. The secondary electron images were acquired in a Zeiss ULTRA plus instrument operating at 3 kV.

The functionalized nanoparticle hydrodynamic diameter was measured in triplicate using a Zetasizer Nano series Nano-ZS ZEN3600 fitted with a red laser light beam (λ = 633 nm) (Malvern Instruments Ltd., UK). The equilibration time was set to 2 min at 25 °C. A suspension of nanoparticles was prepared in water at 1 mg/mL final concentration. The samples were stirred and sonicated when necessary to avoid large aggregates that could interfere with the measurements. The hydrodynamic diameter (size distribution by intensity) represents the average of independent experiments.

The powder X-ray diffraction (PXRD) pattern of the synthesized MSN was acquired in a Rigaku Miniflex II diffractometer with a Cu Kα radiation X-ray source. The dried nanoparticles were mounted on the sample holder and scanned over a 2θ range of 2–10 degrees; step width of 0.01, count time 1, at 30 kV and 15 mA.

The infrared (IR) spectra of MSN were recorded using a Perkin-Elmer Spectrum 100 spectrometer with an attenuated total reflection (ATR) accessory. The spectral range recorded was from 4000 to 600 cm^−1^ (see Supplemental Fig. S2).

The surface area and the pore size distribution of MSN were analysed by nitrogen (N_2_) adsorption–desorption porosimetry. The isotherm was measured on a Gemini VI (Micromeritics Corp. Atlanta, GA) surface analyser. The nanoparticle powder was degassed overnight at 120 °C before analysis. The surface area was determined through the Brunauer–Emmett–Teller (BET) model using the adsorption isotherm data at P/P^0^ from 0.1 to 0.6. The average pore diameter was calculated using the adsorption data applying the Barrett-Joyner-Halenda (BJH) model.

#### Hydrogel characterization

The crosslinked hydrogel was visualized through cryo-SEM in its ‘natural’ hydrated state. The hydrogel sample was cooled in liquid nitrogen and transferred under vacuum into the SEM chamber. The surface of the hydrogel sample was cut to reveal the internal structure.

Hydrogel degradation profile was determined by monitoring dry weight in time. The hydrogels were incubated in PBS at pH 7.4 and 37 °C. At predetermined time points the liquid was removed and the material weight was recorded.

The rheological properties of CX hydrogel were assessed using oscillatory measurements on an AR-2000 Rheometer (TA Instruments) coupled with a parallel plate (diameter 8 mm). The viscoelastic properties were measured at 25 °C in the pre-formed hydrogels at 1 Hz frequency, and the storage modulus G' and loss modulus G’’ were measured as a function of the stress.

### Release studies

#### Release assays from MSN

The loaded MSN were suspended in distilled water (in the case of Safranin-O-loaded MSN-PEG) or PBS at pH 7.4 with 0.5% Tween-80 (PTX-loaded MSN-PEG) and kept under stirring at 400 rpm. At predetermined time points, the sample was centrifuged and the supernatant was collected for analysis.

The stimulus, 10 mM glutathione (GSH), was applied at the beginning of the release assays to the drug-loaded MSN (t = 30 min) and the Safranin-O-loaded MSN (t = 60 min). Following supernatant collections, PBS at pH 7.4 with 10 mM GSH was added to keep the GSH concentration constant. Non-functionalized MSN and MSN in solution without GSH were used, respectively, as positive and negative controls for the drug release assays. Safranin-O release was monitored by UV spectroscopy at 520 nm, and PTX release was monitored by HPLC as previously described^25,31^.

#### Release assays from the hydrogel

The CX hydrogel was prepared by dissolving the polymer in water for a final concentration of 4% w/v after mixing with the crosslinker, as described above. Once the polymer was completely dissolved, 1 mg MSN-PEG was added to 25 μl polymer solution. The crosslinker solution was prepared separately in water (24.3 mg/ml) and mixed with the hydrogel/nanoparticle dispersion. The mixture (50 μl) was added to a 1 mL syringe tip as a mould to have the final hydrogel pieces used in the release assays.

To start the release, 0.2 mL PBS was gently added to the insert containing the material (hydrogel + MSN-PEG) and 1 mL PBS was added to the bottom of the well. At predetermined time points, 1 mL PBS was collected from the bottom of the well and the same amount of fresh PBS was added back.

The collected samples were kept in the freezer until the concentration of nanoparticles was measured by Nanoparticle Tracking Analysis (NTA) using a Nanosight NS300 instrument. The aliquots from the hydrogel release were placed in the Nanosight directly or after dilution when needed, and the laser source was applied to visualize the particles. Each sample was measured three times and a mean percentage release ± Standard Error of the Mean (SEM) is reported.

For the GlioGel release assays, the final formulation containing the target doses of 3 mg TMZ/ mL hydrogel and 20 mg MSN-PTX-PEG/mL hydrogel (5.4 mg PTX) was placed in the insert and PBS at pH 7.4 was added. At predetermined time points, 1 ml aliquot was taken and replaced with fresh PBS. The TMZ release profile from GlioGel was measured through HPLC following a previously described method (mobile phase: 10% ACN/90% water (v/v); flow rate: 1 mL/min; wavelength detection: 316 nm)^33^.

### In vitro cell assays

#### Cell culture conditions

U-87 MG human glioblastoma cell line, acquired from ATCC (https://www.atcc.org/products/htb-14), was cultured in Minimum Essential Medium Eagle (MEM) (Sigma-Aldrich, Ireland) supplemented with 10% FBS and 1% l-glutamine under a humidified atmosphere of 5% CO_2_ at 37 °C.

U-87 spheroids were grown in vitro using a liquid overlay system on the Nunclon Sphera low-attachment surface 96-well plate (ThermoFisher Scientific) using the same media as above, and these 3D cultures were kept under a humidified atmosphere of 5% CO_2_ at 37 °C. The spheroids were grown for 2 days before treatment.

#### Cell viability

For viability tests, the cells were seeded on 24-well plates in an initial density of 25,000 cells/well and left to attach for 24 h. Subsequently, the cells were treated with increasing concentrations of empty MSN or MSN-PEG, free PTX, MSN loaded with PTX (MSN-PTX), or MSN-PEG loaded with PTX (MSN-PTX-PEG). The same cells growing in media without any treatment were used as control. PTX was solubilized in DMSO and the same proportion (1%) of this diluent was added in all treatments.

Cell viability was evaluated using the Cell Counting Kit-8 (CCK-8) (Dojindo, Europe) after 72 h. The reagent was directly mixed with fresh media and added to the cells for 1 h at 37 °C. After that, the absorbance at 450 nm was read. The viability was plotted as the percentage (%) of absorbance normalized for the controls. The relative cell viability values were compared with the untreated cells and all the other treatment groups.

The spheroids grown for 2 days were treated with different concentrations of free TMZ, MSN-PEG, MSN-PTX-PEG and a mix of free TMZ + MSN-PTX-PEG for 72 h. The spheroids viability was measured by the acid phosphatase assay^34,35^ and plotted as a percentage (%) of absorbance at 405 nm normalized for the control (spheroids growing without treatment). The spheroids diameter was monitored on day 2 (before treatment) and day 5 (after treatment), and the growth ratio between those two measurements was calculated.

#### Flow cytometer analysis

To monitor the nanoparticle internalization by U-87 glioblastoma cells, fluorescently labelled nanoparticles were used. The cells were seeded on 6-well plates in an initial density of 50,000 cells/well and left to attach for 24 h. Subsequently, the cells were treated with MSN-FITC or MSN-FITC-PEG (50 µg/mL). The same cells growing without any treatment were used as a control. After 24 h, the medium was removed, and the cells were harvested with trypsin by centrifugation and then resuspended in PBS. The suspension was analysed in a BD Accuri C6 Plus flow cytometer (BD Biosciences) before and after the addition of 50 µl trypan blue, as a FITC quenching agent^36,37^. Three experiments were performed in triplicates and 10,000 events per sample were recorded (Supplemental Fig. S3). The percentage of fluorescent cells after trypan blue addition was plotted and analysed in GraphPad Prism.

#### Confocal fluorescence microscopy

The nanoparticle internalization on 2D cultured cells was analysed by confocal microscopy. The cells were seeded on µ-slide 8 well-chambered coverslips (Ibidi) in an initial density of 12,000 cells/well and left to attach for 24 h. Subsequently, the cells were treated with MSN-FITC or MSN-FITC-PEG (50 µg/mL). The same cells growing without any treatment were used as a control. After 24 h, the non-internalized nanoparticles were removed, the cells were fixed with 10% formalin, and phalloidin and Hoechst 33,342 solution were used to stain the actin cytoskeleton and cell nucleus, respectively. The cells were imaged at a Leica SP8 confocal inverted microscope.

The nanoparticle penetration on 3D spheroids was also analysed by confocal microscopy. The spheroids were grown on the Nunclon Sphera low-attachment surface 96-well plate (ThermoFisher Scientific) for 2 days. Subsequently, the spheroids were treated with 100 μg/mL MSN-FITC or MSN-FITC-PEG. After 24 h, the spheroids were fixed with 10% formalin and stained with phalloidin and Hoechst 33,342 solution. Then, 15 to 20 image stacks (5 μm) of each spheroid were taken at a Leica SP8 confocal inverted microscope. The area of nanoparticles in the spheroids and the total area of the spheroid were estimated using ImageJ software. Then, the proportion of nanoparticles area in relation to the spheroid total area was calculated and plotted as mean ± SEM.

### In vivo study

Our study is reported in accordance with ARRIVE guidelines, with the exception of the sample size calculation. The experimental protocols employed in this study were approved by Trinity College Dublin Animal Research Ethics Committee and the Health Products Regulatory Authority (HPRA) in Ireland (project authorisation number AE 19136 /P102), and all experiments were performed in accordance with relevant guidelines and regulations. The use and treatment of mice throughout the study was performed according to the Three R’s guidelines for ethical animal testing. Female immunodeficient CIEA NOG mice® (22 ± 3 g) were housed in groups of 4 and kept under standard housing conditions at a constant temperature (20 ± 2 °C) and standard lighting conditions (12 h light: 12 dark cycles). Food and water were available ad libitum.

#### Orthotopic U-87 MG human glioblastoma tumour model

To establish a GBM tumour, mice were anaesthetized using 2% isoflurane and maintained in an anaesthetized state at 1.5%. Using a stereotactic frame, the scalp hair at the surgical site was removed, an incision along the midline was made in the right frontal lobe, and a small hole was drilled to expose the brain (coordinates from bregma: 0.5 mm posterior and 2.5 mm lateral). 3 × 10^4^ U-87 GBM tumour cells in complete cell culture media (2.5 μl) were injected into the brain at a depth of 1.5 mm. The wound was closed with 4/0 interrupted simple sutures and cleaned with alcohol and iodine^38^.

To monitor tumour growth, the animal’s brain was scanned at days 14, 21 and 26 after cell injection. All magnetic resonance imaging (MRI) was carried out on a dedicated rodent Bruker Biospec system (Bruker Biospin, Germany) with a 7 Tesla magnet and a 30 cm diameter bore, equipped with a 20 cm actively shielded gradient system. A pair of actively decoupled 12 cm Helmholtz transmit, and 3 cm surface quadrature receive coils (Bruker Biospin, Germany) were used for signal transmission and reception respectively. Coronal brain images were acquired using a two‐dimensional multislice T1-weighted FLASH, 10 averages with TE = 2.461 ms, TR = 326.837 ms, a flip angle of 60°, a field of view of 2 cm × 2 cm (voxel size: 0.156 × 0.156 × 0.5 mm^3^). For each animal, 30 consecutive coronal sections of 0.5 mm thickness were acquired (interslice distance of 0.55 mm). The total acquisition time was 4 min 41 s 79 ms. Images were reconstructed with Paravision 6.0 software (Bruker Biospin, Germany). The tumour volumes were calculated using MIPAV software by manually drawing the region of interest (ROI). The curve was fitted with an exponential growth equation.

#### Resection surgery and hydrogel implantation

The presence and size of the tumour were evaluated by MRI around day 19 after tumour cell injection. The resection surgery modified from Sweeney et al.^39^ and Bianco et al.^38^ was performed on day 23 ± 2 based on the size of the tumour evaluated by MRI. Briefly, a mouse was anaesthetized using 2% isoflurane and maintained in an anaesthetised state at 1.5%. Afterwards, it was transferred to a stereotactic frame, the skull was exposed and a hole was drilled at the same location of the injection site. A Pasteur pipette connected to a vacuum pump was used to gently remove the tumour. After the resection, the animals were randomly divided into two groups and hydrogel was administered in the tumour cavity. Mice were randomly assigned to treatment groups without regard to any other variables. The control group received an empty hydrogel (4–5 μl; n = 5), and the treatment group received the GlioGel formulation (4–5 μl), containing the dose of 0.6 mg/kg free TMZ and 0.3 mg/kg PTX (loaded in MSN-PEG) (n = 4). To repair the cranial defect, bone wax was placed in the hole, the skin was closed with 4/0 interrupted simple sutures and cleaned with alcohol and iodine.

#### Animal welfare

Animals were monitored daily for any sign of pain and/or distress during the experiment period. Each animal was closely monitored following surgery to detect immediate adverse effects and to ensure full recovery after anaesthesia. All daily monitoring, scoring, weights, supportive care and treatment were recorded in score sheets. In circumstances where profound discomfort was observed, or when a mouse has exceeded 20% weight loss, the animal was humanely euthanized by CO_2_ inhalation.

#### Histological analysis

The animal brain was harvested when the mouse died or reached a humane endpoint and was euthanized, or at the end of the experiment, 5 weeks after the resection surgery. The brain was fixed in 4% paraformaldehyde at 4 °C for approximately 24 h and dehydrated in 20% sucrose in PBS at 4 °C until sectioning in a vibratome. Slices between 50 and 90 μm were cut, mounted in slides and stained with haematoxylin/eosin. The slides were imaged with an Olympus BX51 upright microscope.

### Statistical analysis

All the experiments were performed in triplicate (plotted as mean ± SEM) and the statistical analysis was made using GraphPad Prism. Analyses of variance (ANOVA) followed by Tukey’s post-test (comparing all treatment groups) or Dunnett’s post-test (treatment groups against the controls) were performed where relevant. Data were considered significant when P < 0.05 (*), P < 0.01 (**) or P < 0.001 (***).

For the in vivo experiment, measurements and experimental evaluations were carried out blind. Of the 20 mice in the experiment, 6 mice were not included in the final analysis: one died during surgery, one did not develop a tumour after cell injection and four experienced injection failure. The treatment groups were as follows: tumour (no treatment) (n = 5), tumour + resection + empty hydrogel (n = 5), tumour + resection + GlioGel (n = 4). The Kaplan–Meier survival curves were analysed with a log-rank test (Mantel-Cox test). The multiple comparisons were corrected by the Bonferroni threshold with a significance level set at 0.05. The tumour volume after resection (recurrence) was compared through a Mann Whitney test.

### Ethics approval and consent to participate

The in vivo protocols employed in this study were approved by the local Animal Research Ethics Committee in Trinity College Dublin (Ireland) and the Health Products Regulatory Authority (HPRA) in Ireland.

## Results and discussion

**Figure 2:**
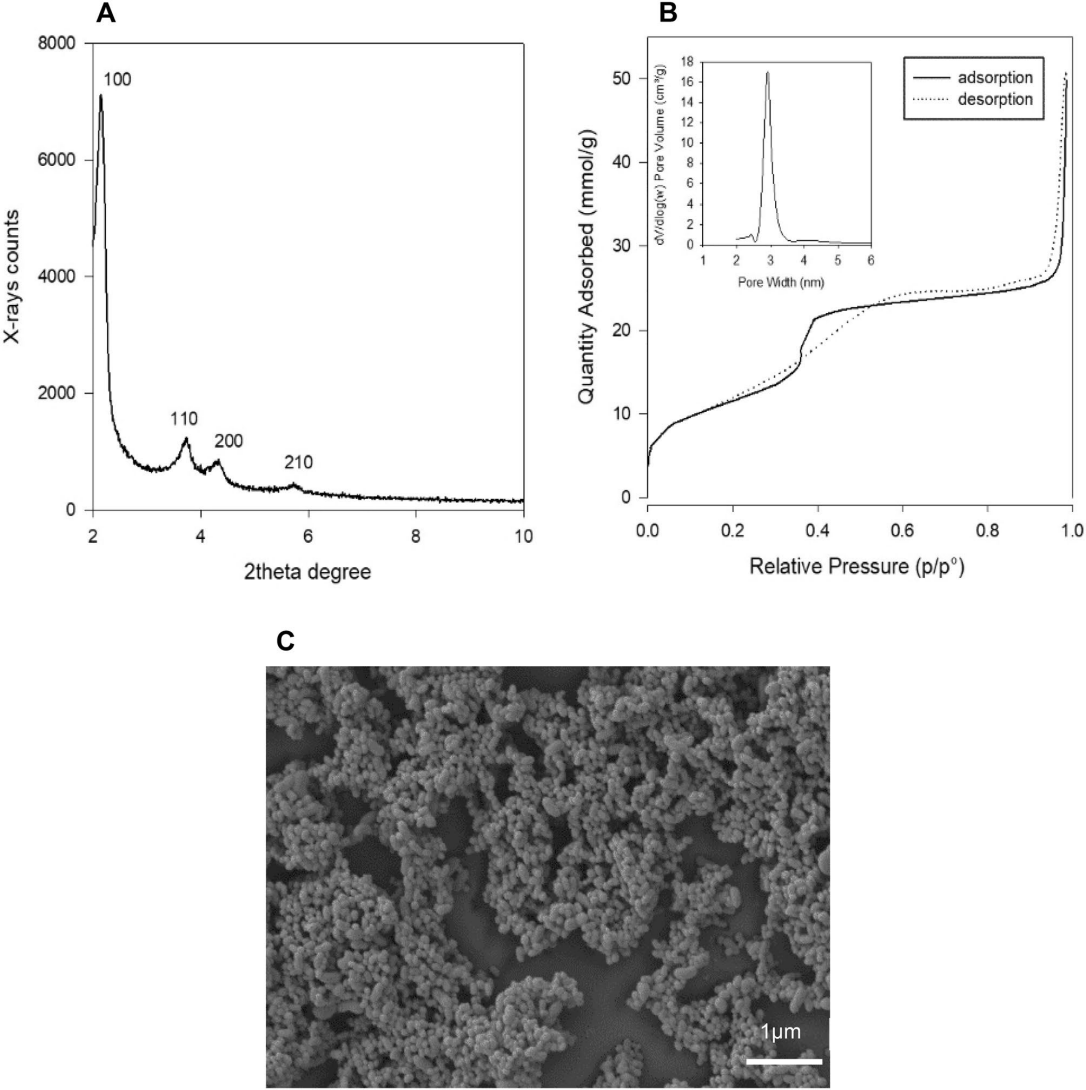
MSN structural and textural characterization. The nanoparticles were characterized to determine the hexagonal pore organization, pore volume and size, surface area and morphology. (**A**) Powder X-ray diffraction pattern at low angle of MSN. (**B**) N_2_ adsorption–desorption porosimetry isotherm, and pore size distribution (inset). (**C**) Scanning electron microscopy (SEM) image of the nanoparticles.

As-synthesized mesoporous silica nanoparticles (MSN) after surfactant extraction showed the characteristic structural features of an MCM-41 type material (Fig. 2A). The powder X-ray diffraction pattern showed four diffraction peaks, indexed as (100), (110), (200), (210) at 2.1°, 3.6°, 4.2° and 5.7° respectively. These peaks confirmed a hexagonal organization of the pores in the amorphous nanoparticle network. As shown by the nitrogen sorption porosimetry (Fig. 2B), the nanoparticles show a characteristic type IV isotherm with a high surface area of 944 m^2^/g and an average pore diameter of 2.8 nm. They also showed a uniform distribution in size and spherical morphology, as observed by scanning electron microscopy (SEM) (Fig. 2C).

Together with their reported biocompatibility^40,41^, the high surface area of MSN is among the main features of these nanocarriers, since it allows a high loading of therapeutics. Additionally, their surface chemical characteristics enable them to be functionalized with different patterns^42^. These characteristics allow the design of a responsive delivery system that provides a triggered drug release in the presence of higher concentrations of reducing agents, such as intracellular GSH.

**Figure 3:**
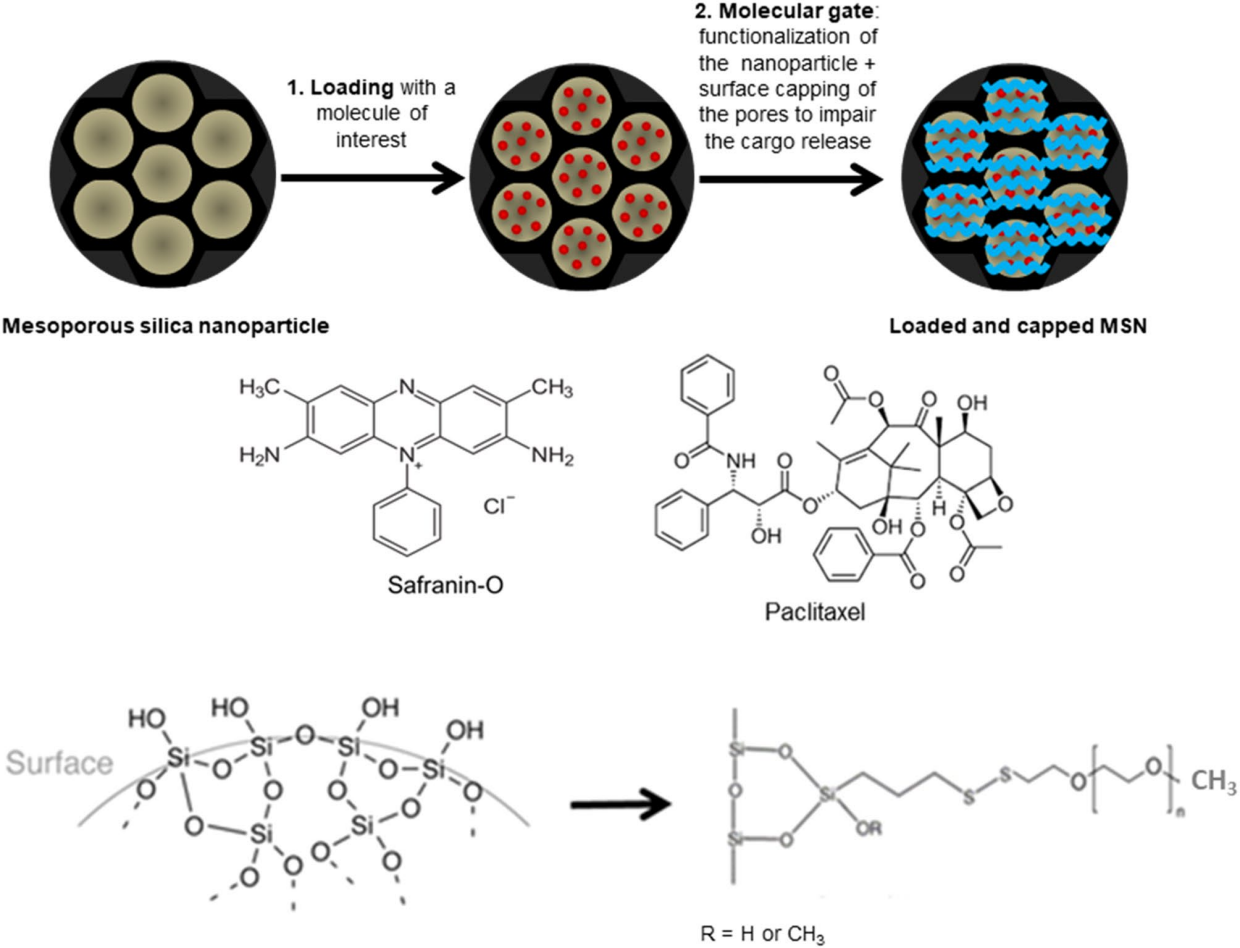
MSN loading and surface functionalization process. The nanoparticles were loaded with either a dye model molecule (Safranin-O) or a chemotherapeutic drug (paclitaxel). Then, the surface of the MSN was functionalized with PEG, which was attached through a disulphide bond building a redox-responsive molecular gate.

Following surfactant extraction, the MSN were loaded with either Safranin-O, a model dye molecule, or PTX. To build a redox-responsive gate on the MSN surface that would minimize drug release while the MSN are contained within the GlioGel formulation, the loaded nanoparticles were functionalized with PEG through a disulphide bond (Fig. 3), sensitive to the intracellular reducing environment. The PTX loading process yielded an encapsulation efficiency of 67% and a loading capacity of 18%.

**Figure 4:**
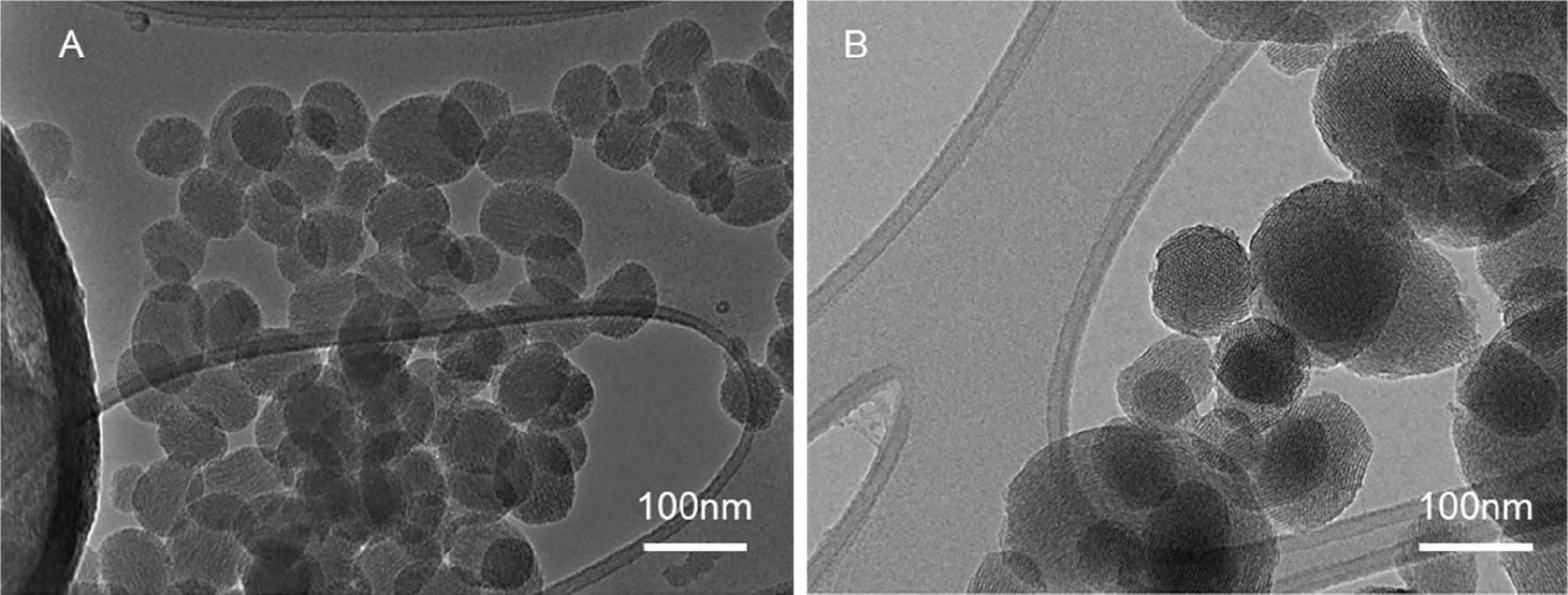
The morphology of the MSN did not significantly change after functionalization with PEG. (**A**) TEM image of MSN. (**B**) TEM image of MSN-PEG.

The functionalization did not affect the morphology of the nanoparticles (Fig. 4). The average size of the non-functionalized nanoparticles as observed by TEM was 137 nm. After the addition of PEG, the size observed by TEM did not significantly change (133 nm), and a hydrodynamic diameter of 193 nm (PDI 0.42) was found when MSN-PEG was in aqueous dispersion (Fig. S1). The presence of PEG on the surface of the nanoparticles was confirmed by Fourier-transform infrared spectroscopy (FTIR) (Fig. S2), where the bands corresponding to C–H stretching were identified after functionalization.

**Figure 5:**
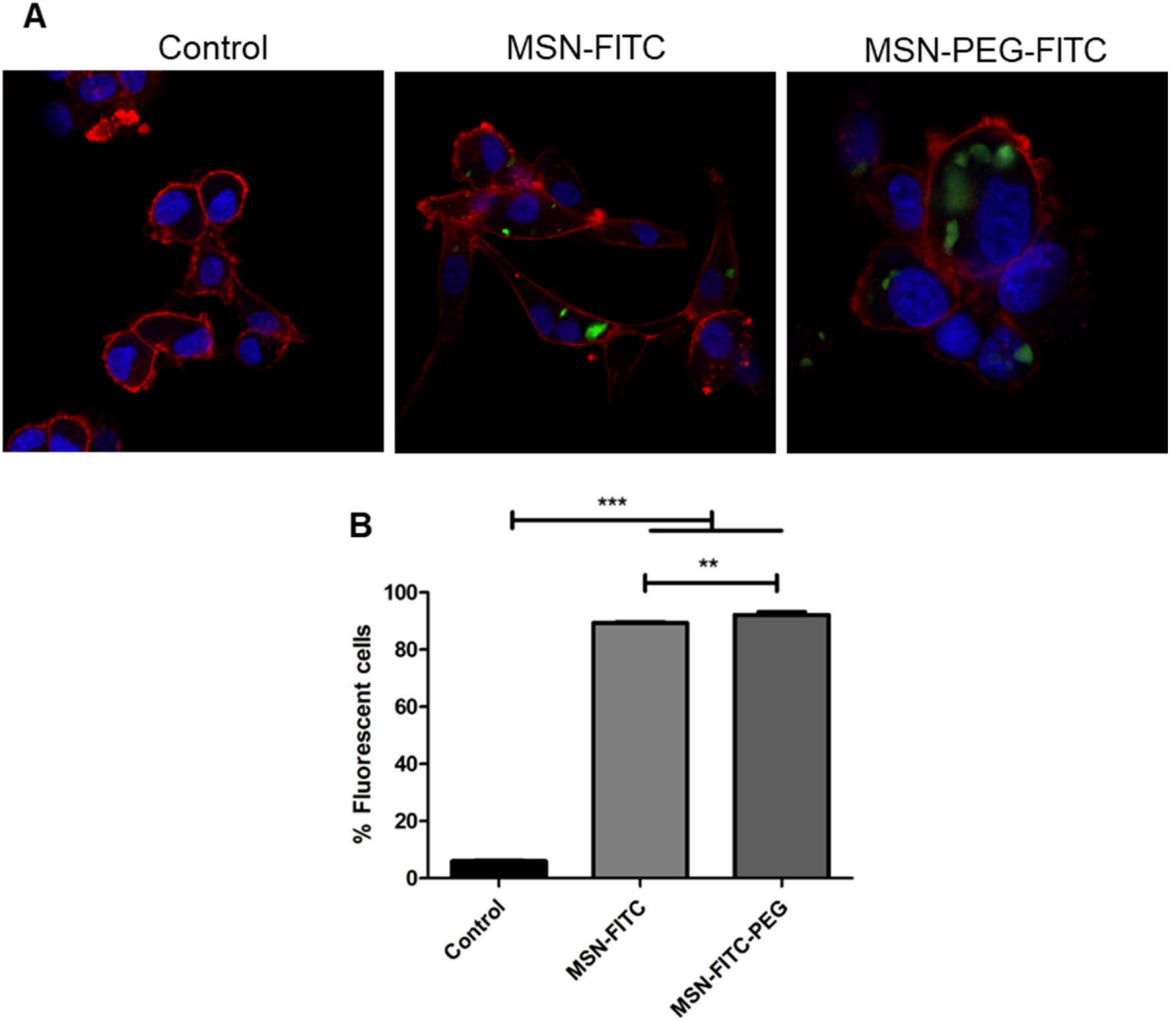
The MSN are internalized on U-87 glioblastoma cells. (**A**) Confocal Fluorescence microscopy of U-87 cells after 24 h treatment with 50 µg/ml MSN-FITC and MSN-FITC-PEG. (**B**) Fluorescent cells detected by flow cytometry after 24 h treatment with 50 µg/ml MSN-FITC and MSN-FITC-PEG. Control = cells without the addition of nanoparticles. Statistical test: One-way ANOVA with Tukey’s post-test comparing all treatments. P < 0.05 (*), P < 0.01 (**) or P < 0.001 (***).

Nanoparticle internalization results evidenced that cells incubated with MSN-FITC-PEG for 24 h presented a higher presence of nanoparticle spots (green) compared to MSN-FITC, as observed by confocal microscopy (Fig. 5A). While only 6% of the cells presented a background fluorescent signal (controls without nanoparticles), 92% of cells were fluorescent when treated with MSN-FITC-PEG, and 89% when treated with MSN-FITC (Fig. 5B), which indicates effective nanoparticle internalization in both cases. Similar results were observed by flow cytometry, with 93% of fluorescent cells when treated with MSN-FITC-PEG and 91% fluorescent cells when treated with MSN-FITC (Fig. S3a–c).

These results show that PEGylation did not impair nanoparticle internalization by U-87 cells after 24 h in contact with the cells. This is supported by previous observations with PEGylated PLGA-based nanoparticle internalization on HeLa cells verified by flow cytometry (up to 24 h incubation)^31^, brain endothelial cells uptake of PEGylated silica nanoparticles observed by confocal fluorescence microscopy (4 h incubation)^43^ or MSN functionalized with PEG through a disulphide bond internalized in MCF7 cells after 24 h incubation^44^.

**Figure 6:**
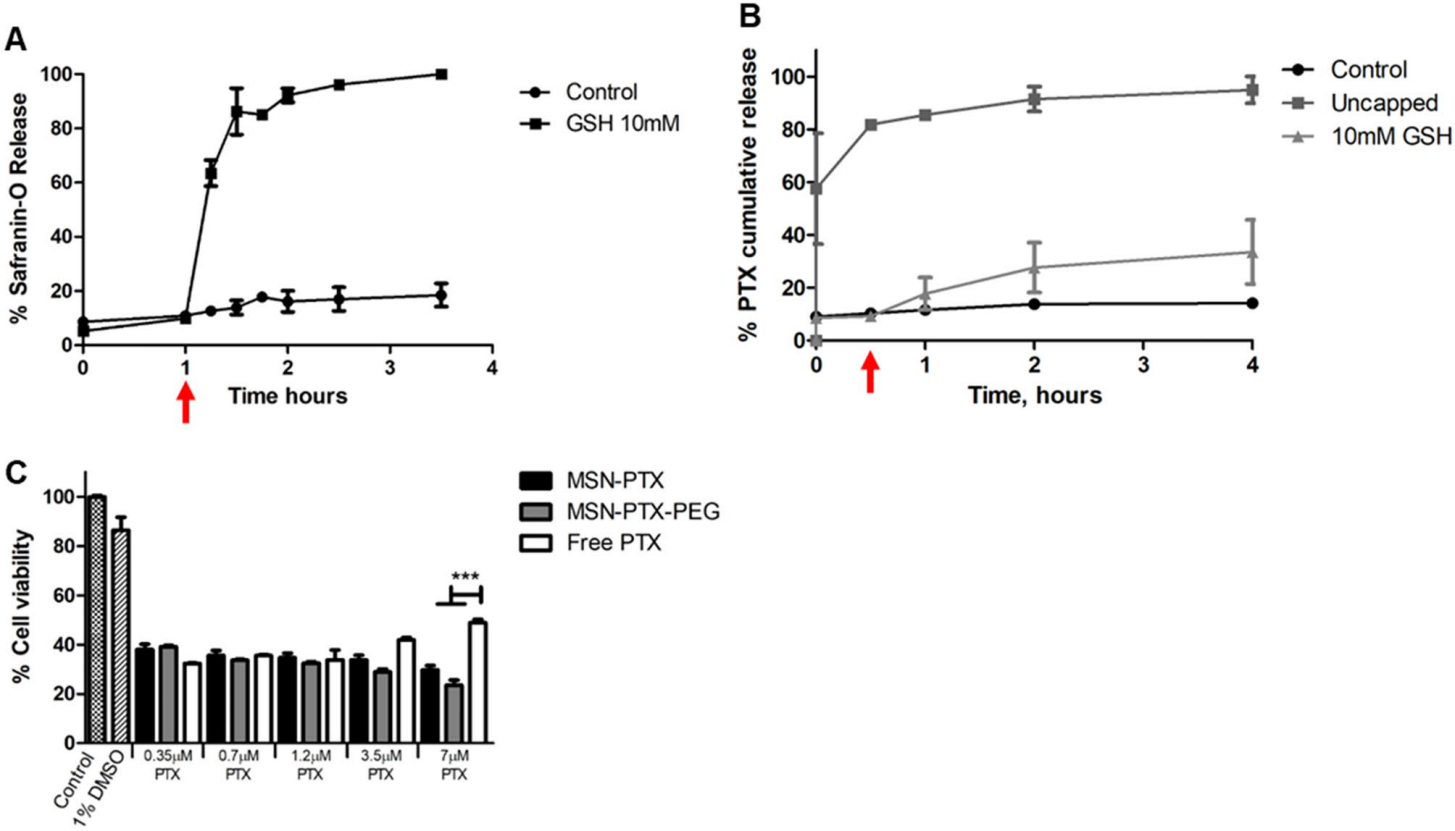
The successful formation of the responsive gate was confirmed by the release assays. Release from MSN-PEG was triggered after the addition of GSH as a reductive stimulus. (**A**) Safranin-O release profile. (**B**) PTX release profile. The results, presented as mean ± SEM of at least 2 independent measurements, were normalized for the highest amount released in the assay. Red arrows indicate the time of 10 mM GSH addition on the triggered release group (at 1 h for Safranin-O and at 30 min for PTX release assays). (**C**) Comparison of U-87 cell viability upon treatment with free PTX, MSN-PTX and MSN-PTX-PEG with equivalent drug concentrations. PTX was solubilized in DMSO and the same proportion (1%) of this diluent was added in all treatments. Statistical analysis: Tukey’s post-test comparing all treatments at equivalent drug concentration. P < 0.001 (***).

The successful formation of a responsive gate on the MSN pores after PEGylation was confirmed by the release assays presented in Fig. 6A,B. The release of Safranin-O was used to test the effectiveness of the molecular gate in a preliminary assay. As observed in Fig. 6A, the release of Safranin-O was triggered after the GSH addition. This effect was reproduced in the PTX release study from MSN-PEG (Fig. 6B) as compared to a negative control without the addition of GSH, and the non-PEGylated MSN (positive control). The basal release from MSN-PEG was around 15% of the maximum release over the time of the experiment, and these nanoparticles released almost 40% when the stimulus was added. This type of redox-responsive molecular gates have been previously described and applied against a range of tumours^25,44,45^.

The effect of MSN, MSN-PEG, MSN-PTX, MSN-PTX-PEG and free PTX was evaluated on U-87 cells (Fig. 6C and Supplemental Fig. S4). Equivalent PTX concentrations from 0.35 to 1.2 µM had very similar effects on the cells when the drug was free or loaded into either MSN or MSN-PEG, decreasing the viability to 32% (free PTX), 35% (MSN-PTX) and 32% (MSN-PTX-PEG). The fact that MSN-PTX-PEG achieved similar GBM cell killing activity as MSN-PTX confirms their cell internalization and the activation of the redox-responsive release in contact with the reducing environment of the cells. Importantly, the free drug had a reduced effect at higher concentrations while both MSN-PTX and MSN-PTX-PEG further increased their cell-killing activity. A similar loss of cytotoxic effect at higher concentrations of PTX at 24 h, 48 h and 72 h was previously reported for different cancer cell lines^46^. This effect was attributed to both the phase of cell growth (plateau or exponential) and the diluent (Cremophor) used in the study. In our experiment, we hypothesised that the loss of cytotoxicity may be due to a combination of a plateau phase of growth and drug resistance developed by the cells. In this case, the incorporation of PTX into the nanoparticles showed an advantage over the free drug.

Having developed a responsive nanoparticle able to carry a chemotherapeutic drug and exert an in vitro cytotoxic effect on U-87 GBM cells, we advanced into a proof-of-concept of the GlioGel formulation combining a drug-loaded injectable hydrogel and MSN. For this purpose, we prepared and characterized chemically crosslinked hydrogels based on poly(1-(acetonylamino)-2-methyl-2-propen-1-one), and we analysed the incorporation and release of MSN-PEG and free drug (TMZ) from the hydrogels.

**Figure 7:**
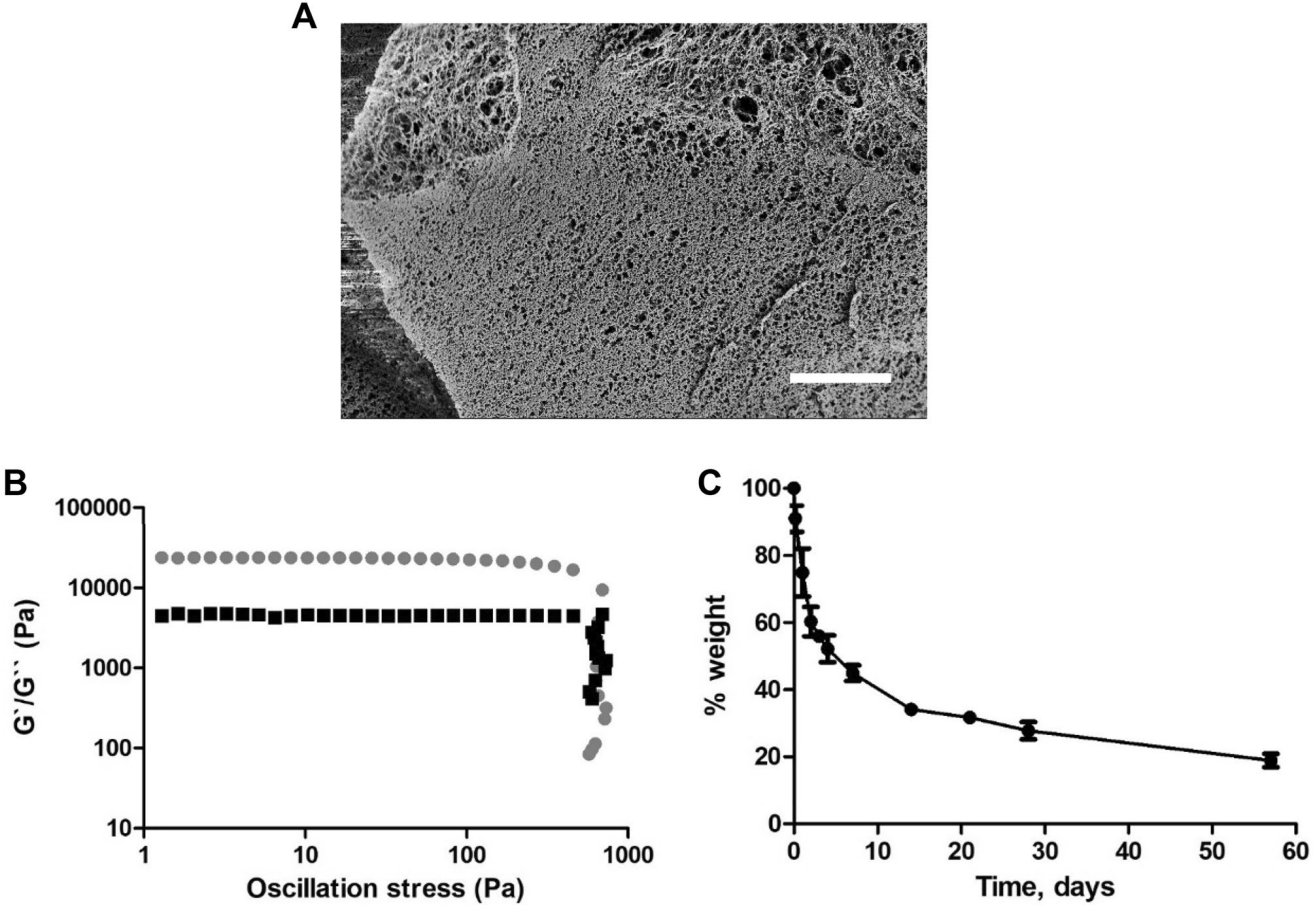
Crosslinked (CX) hydrogel characterization. (**A**) Crosslinked hydrogel structure by Cryo-SEM (scale bar = 5 µm). (**B**) CX hydrogel rheology measurements (amplitude sweep). (**C**) Crosslinked hydrogel (40 mg/ml) degradation in PBS pH 7.4 at 37 °C. Results are presented as mean ± SD of 3 independent measurements.

The CX hydrogel forms upon the addition of a crosslinker becoming an opaque white solid with the shape according to the mould used in the hydrogel preparation. Cryo-SEM imaging of the CX hydrogel indicates a highly porous network (Fig. 7A). Moreover, the rheology analysis indicates that it has a gel-like or solid structure^47^, since the storage modulus (G’) is greater than the loss modulus (G’’) (Fig. 7B) in the 1–350 Pa range. The CX hydrogel is, therefore, a viscoelastic solid material. This solid-like behaviour is comparable to the brain tissue in which the CX hydrogel is intended to be implanted, although it presents a higher rigidity^48,49^. This is represented by the storage modulus of 24 × 10^3^ Pa. However, this higher rigidity of the CX hydrogel is not expected to compromise the GlioGel formulation performance on GBM treatment. Finally, the CX hydrogel presents a sharp degradation profile in the first 7 days, with 55% weight loss, and more than 80% weight is lost in 58 days (Fig. 7C).

**Figure 8:**
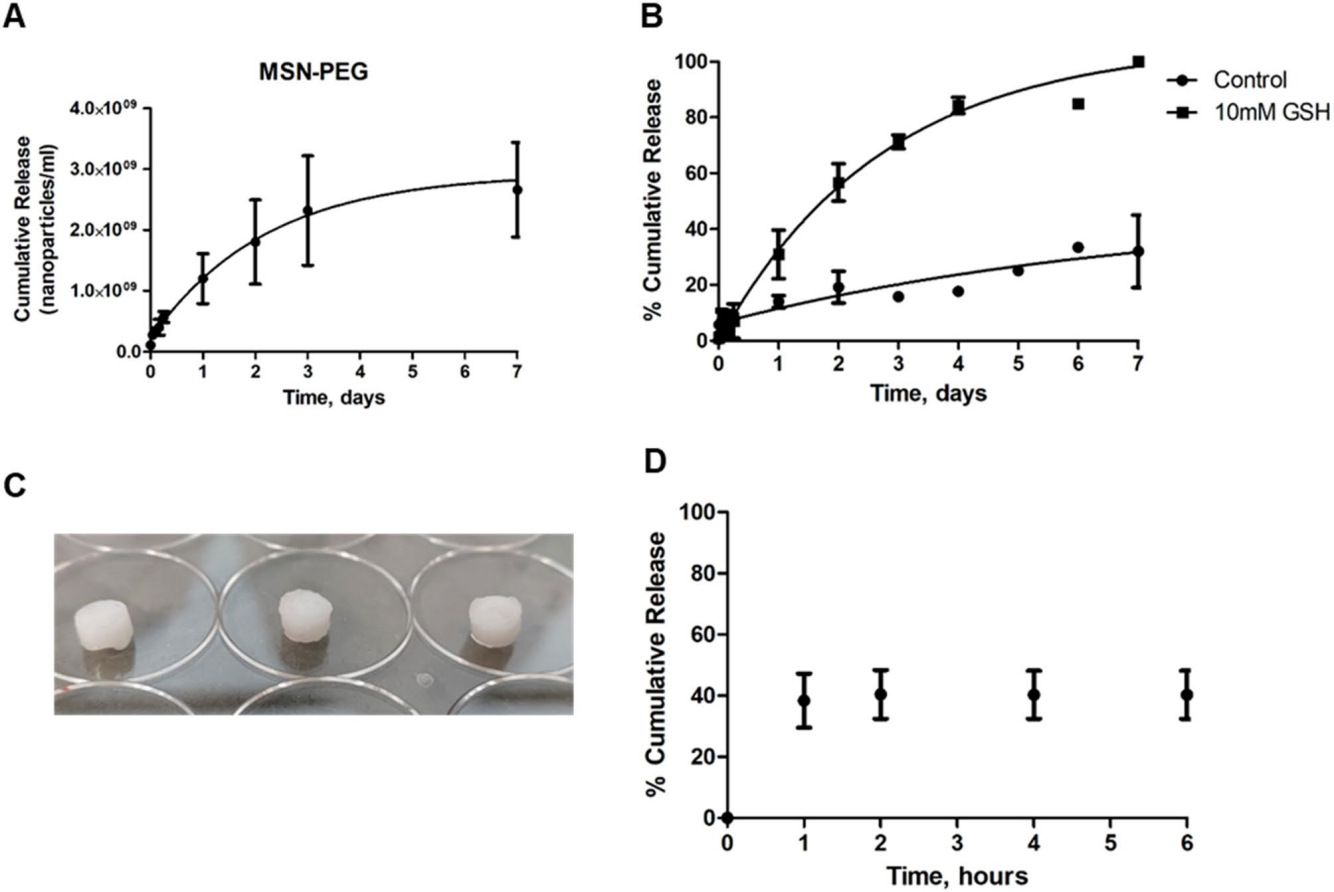
Crosslinked hydrogel release assays. (**A**) MSN-PEG release profile from the CX hydrogel. (**B**) Safranin-O release from MSN-PEG incorporated into CX hydrogel. (**C**) The CX hydrogel (50 µL) was formulated with 150 µg TMZ and 1 mg MSN-PTX-PEG (corresponding to 270 µg PTX) to form GlioGel. (**D**) TMZ release from GlioGel formulation in the first 6 h.

The release of MSN-PEG from the CX hydrogel over 7 days as estimated by NTA is shown in Fig. 8A, in which both the high porosity of the CX hydrogel and its degradation profile may be contributing factors. To test the integrity of the redox-responsive molecular gate following hydrogel formation, the release of Safranin-O from MSN-Safranin-PEG incorporated into the CX hydrogel was monitored in the presence and absence of the GSH trigger. A sustained release of Safranin-O was observed, with a clear effect of the molecular gate when compared to the control (without GSH) over 7 days (Fig. 8B).

The GlioGel formulation (Fig. 8C) comprises the CX hydrogel loaded with free TMZ and MSN-PTX-PEG. A safe dose previously tested for this drug combination treatment in mice^50^ was chosen to be used in the in vivo study. Therefore, considering the loading efficiency of the nanoparticles and the maximum volume of gel injection in the brain of the mice, the GlioGel formulation was designed to contain 3 mg/ml TMZ and 20 mg/ml MSN-PTX-PEG. This formulation showed a burst release of TMZ in the first hours of the release assay (Fig. 8D), ensuring the availability of this drug immediately after GlioGel implantation. Taken together, our release results indicate that a two-step treatment could be achieved from this proposed formulation. In the first step, an immediate release of the free TMZ would combat the remaining cells after surgical resection while, in the second step, the nanoparticles would be released in a sustained manner from the hydrogel and deliver their therapeutic cargo mainly in intracellular reducing environments, such as the one in highly metabolic cancer cells^51,52^.

**Figure 9:**
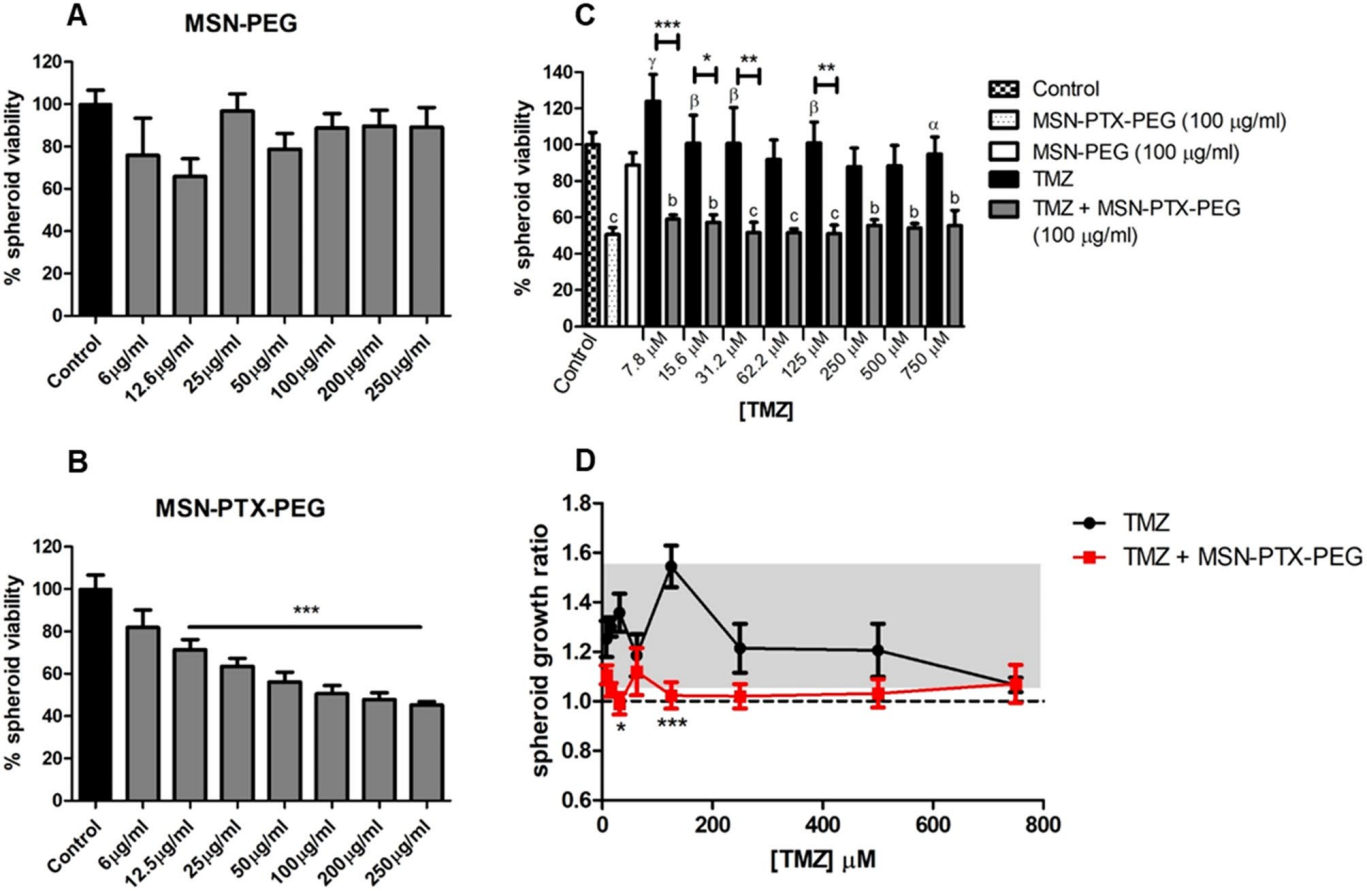
Combination treatment effects on U-87 GBM spheroids. The spheroids were treated for 72 h with different concentrations of (**A**) MSN-PEG; (**B**) MSN-PTX-PEG; and (**C**) TMZ and TMZ in combination with 100 µg/ml MSN-PTX-PEG. (**D**) Spheroids growth ratio after treatment with different concentrations of TMZ and TMZ in combination with 100 µg/ml MSN-PTX-PEG. The grey area corresponds to the growth ratio + /- SEM of control spheroids. Statistical test: One-Way ANOVA for (**A**–**C**) and Two-way ANOVA for (**D**). In (**A**) and (**B**), the treatment groups are compared to the control. In (**C**), differences from the control (spheroids without treatment) are represented as follows: b = p < 0.01; c = p < 0.0001. Differences from MSN-PTX-PEG (100 µg/ml) are represented as follows: α = p < 0.05; β = p < 0.01; γ = p < 0.0001. Differences between free TMZ and combination treatment is represented as follows: *p < 0.05; **p < 0.01; ***p < 0.0001.

Prior to an in vivo evaluation, the drug combination in the GlioGel formulations was tested using a 3D cell culture system, which is considered a more clinically relevant model^53^ when compared to traditional bidimensional cell culture. Therefore, we tested the effect of MSN-PEG, MSN-PTX-PEG, free TMZ and the combination of TMZ + MSN-PTX-PEG in U-87 GBM spheroids. As observed in Fig. 9A,B, MSN-PTX-PEG showed a dose-dependent effect on the spheroids with 100–250 μg/ml of nanoparticles decreasing the viability to 45–50%, while MSN-PEG showed viabilities higher than 80% in the same concentration range.

TMZ alone had a very modest effect on the viability of spheroids for the concentration range tested (Fig. 9C). A mild decrease in viability is observed, with the highest concentration (750 μM) showing a 5% reduction in viability. In contrast, TMZ combined with MSN-PTX-PEG at 100 μg/ml significantly decreased the viability of spheroids compared with TMZ alone. This decrease is comparable to the effect of MSN-PTX-PEG alone (Fig. 9B), indicating that the PTX-loaded nanoparticles are majorly responsible for the treatment efficacy.

**Figure 10:**
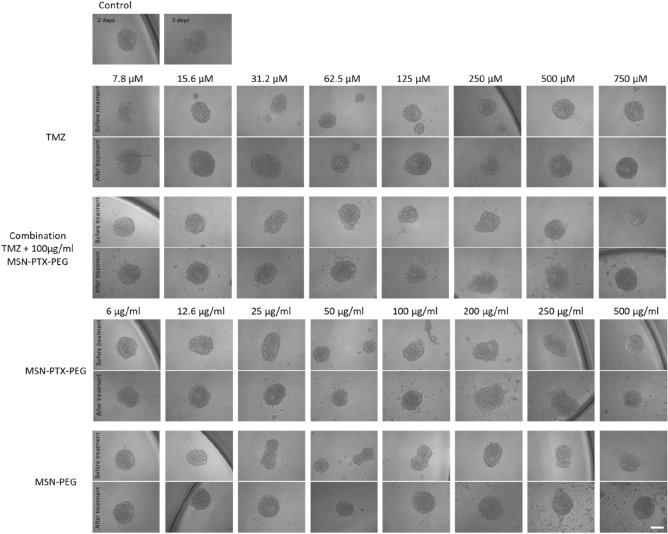
Representative images of U-87 GBM spheroids grown for 2 days and either untreated (upper panels) or treated for 72 h (lower panels) with TMZ, TMZ combined with 100 µg/ml MSN-PTX-PEG, MSN-PTX-PEG and MSN-PEG. Images are representative of three independent experiments (scale bar 200 µm).

As displayed in Fig. 10, the spheroids treated with MSN-PEG were mostly intact and maintained their size and morphology while the ones treated with MSN-PTX-PEG showed signs of a slight disintegration with some detached fragments or cells around the spheroids, and smaller sizes in some cases. The same aspects observed on the appearance of the spheroids after the treatment with MSN-PTX-PEG were found for the combined treatment (TMZ + MSN-PTX-PEG) in the concentration range tested. Higher concentrations of TMZ alone where needed to reach the same growth inhibition effect (Fig. 9D). In all combination ratios, we observed small fragments and individual cells detached from the spheroids. Although this cell detachment was not enough to substantially decrease the size of the spheroids, it inhibited their growth over time.

**Figure 11:**
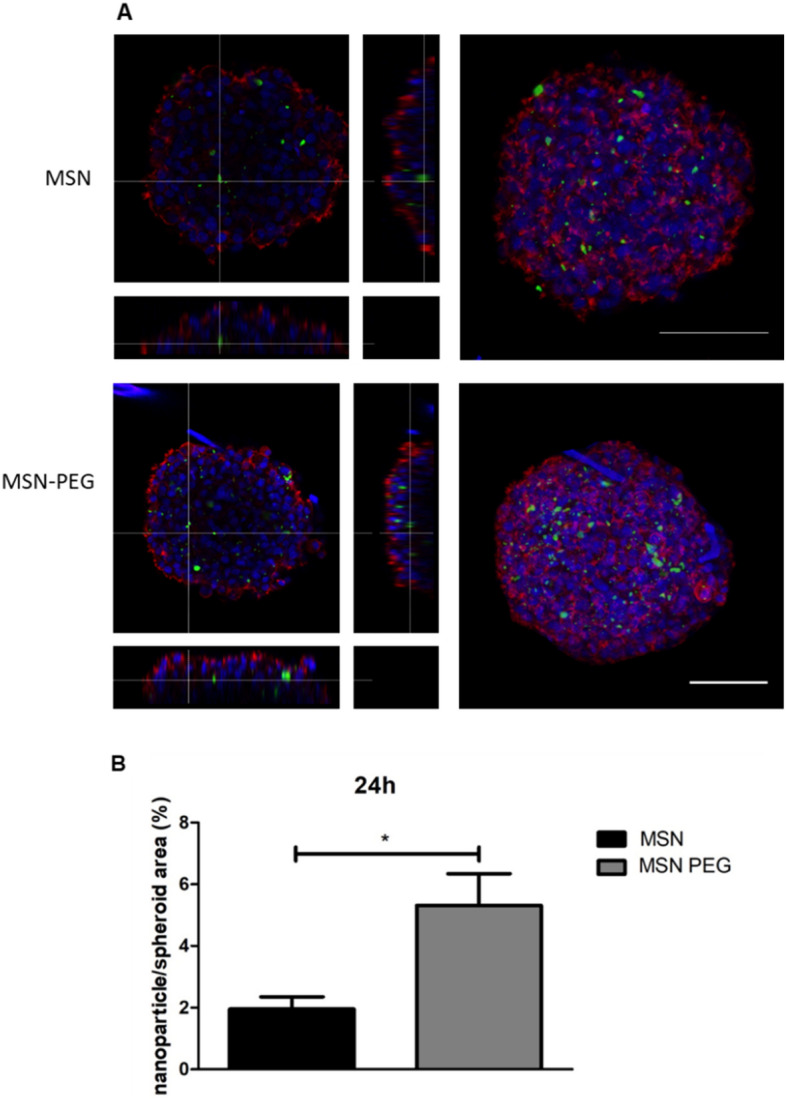
MSN-FITC and MSN-FITC-PEG penetration into U-87 GBM spheroids. (**A**) Left: orthogonal images are the combination of 10 z-stacks of 5 µm each (50 µm from the bottom to the core of the spheroid). Right: combination of all z-stacks (up to 23 stacks). Quantification of nanoparticle (FITC) area on the combined image stacks in relation to the spheroid total area after 24 h. Results are mean ± SEM of three different images. *p < 0.05.

The effect observed when MSN-PTX-PEG are included in the treatment of the spheroids is directly dependent on the ability of the nanoparticles to penetrate them. Nanoparticle PEGylation has been previously shown to enhance penetration of spheroids in the case of polymeric nanoparticles composed of poly(glycerol-adipate)^54^. The modified nanoparticles were reported to penetrate the core of HCT116 colorectal cancer spheroids in a short time frame (4 h) compared to unmodified nanoparticles. In the case of U-87 GBM spheroids, an increased penetration of glycosylated dendrimers compared to unmodified dendrimers was reported by Dhanikula et al.^55^ after 24 h incubation. Moreover, higher penetration of PEGylated polystyrene-based nanoparticles of several sizes (40, 100, 200 nm) into the brain tissue of humans, rats and mice was achieved when compared to non-PEGylated nanoparticles^56^. To assess the role of PEGylation on the silica-based nanoparticles included in the formulation of GlioGel, we compared the ability of MSN and MSN-PEG to penetrate spheroids after 24 h incubation. As represented in Fig. 11, a statistically significant (p < 0.05) difference was observed from the analysis of the confocal microscopy images from both types of nanoparticles. Nevertheless, the images showed that nanoparticles were localized both in the intercellular space and in the cytoplasmic area, which confirms U-87 cell internalization. This observation is in contrast to previous experiments on different nanoscale drug delivery systems, such as the graphene oxide flakes studied by Lázaro et al.^57^, which reported penetration into U-87 spheroids after 24 h, but showed that the material was mainly localized into the extracellular space.

Overall, we can conclude that MSN-PEG manages to penetrate the U-87 GBM spheroids, and that PEGylation not only allows the formation of the molecular gate to control the release of the cargo from the nanoparticles, but also tends to facilitate penetration into the 3D spheroid model leading to growth inhibition upon PTX release.

**Figure 12:**
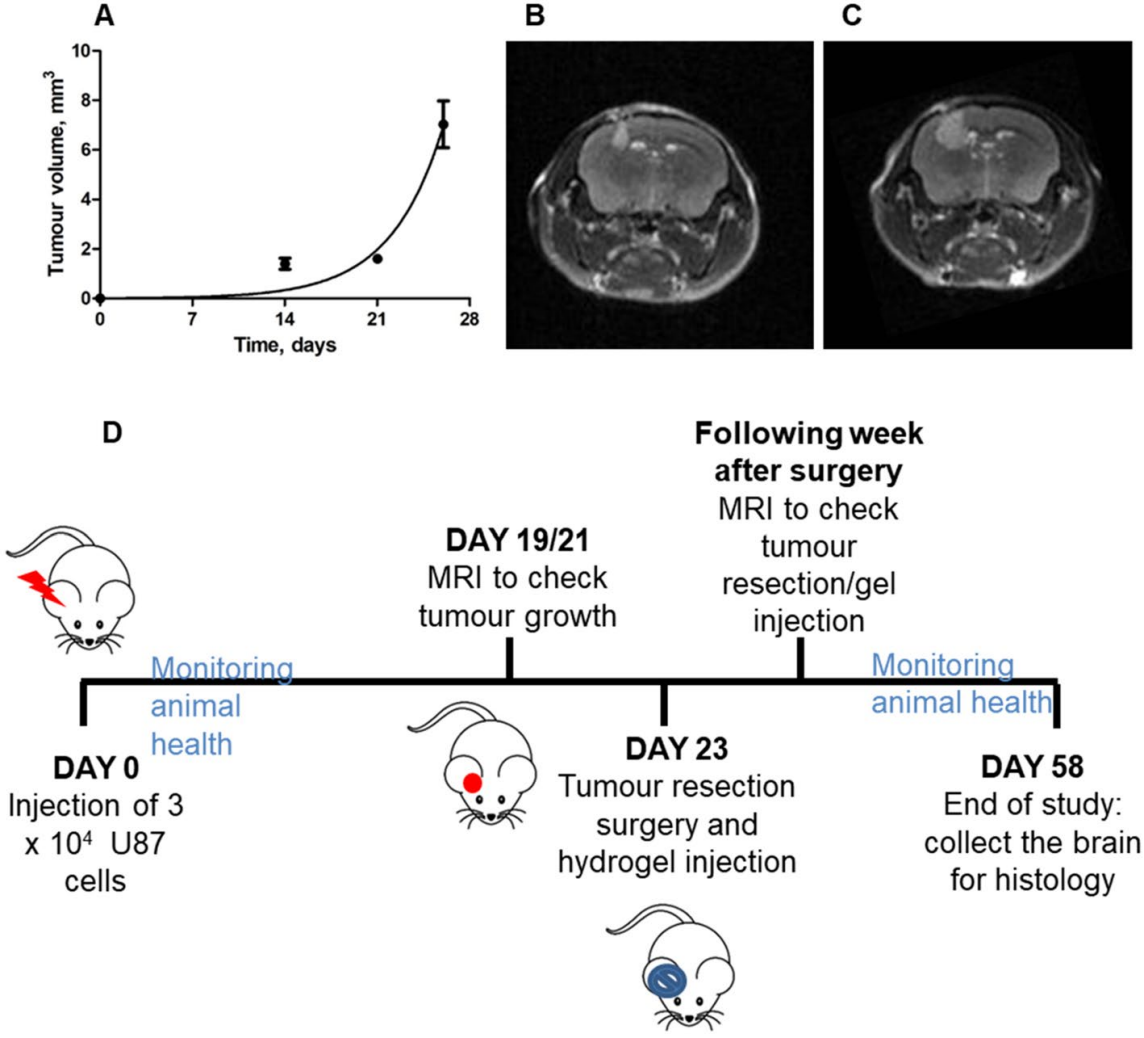
Tumour growth kinetics were monitored by Magnetic Resonance Imaging (n = 5). (**A**) Tumour growth curve fitted with an exponential growth model. (**B**) MRI image of tumour at day 21. (**C**) MRI image of tumour at day 26 after U-87 cell injection. (**D**) Schematic of in vivo protocol.

To evaluate the GlioGel formulation, we used an orthotopic U-87 tumour xenograft model in mice, monitoring the tumour growth by MRI. Based on the tumour growth rate, represented in Fig. 12A, resection surgery was planned around day 23, when the tumour size reached 5 mm^3^. Figure 12B,C show representative MRI images of the tumour in the brain of an untreated mouse at days 21, with a tumour volume of 1.6 mm^3^, and 26, with a tumour volume of 7 mm^3^, respectively. GlioGel was injected directly after tumour resection, and animal health and treatment effects were monitored up to 5 weeks following surgery. Signs of tumour recurrence were monitored by MRI one week after surgery (Fig. 12D).

**Figure 13:**
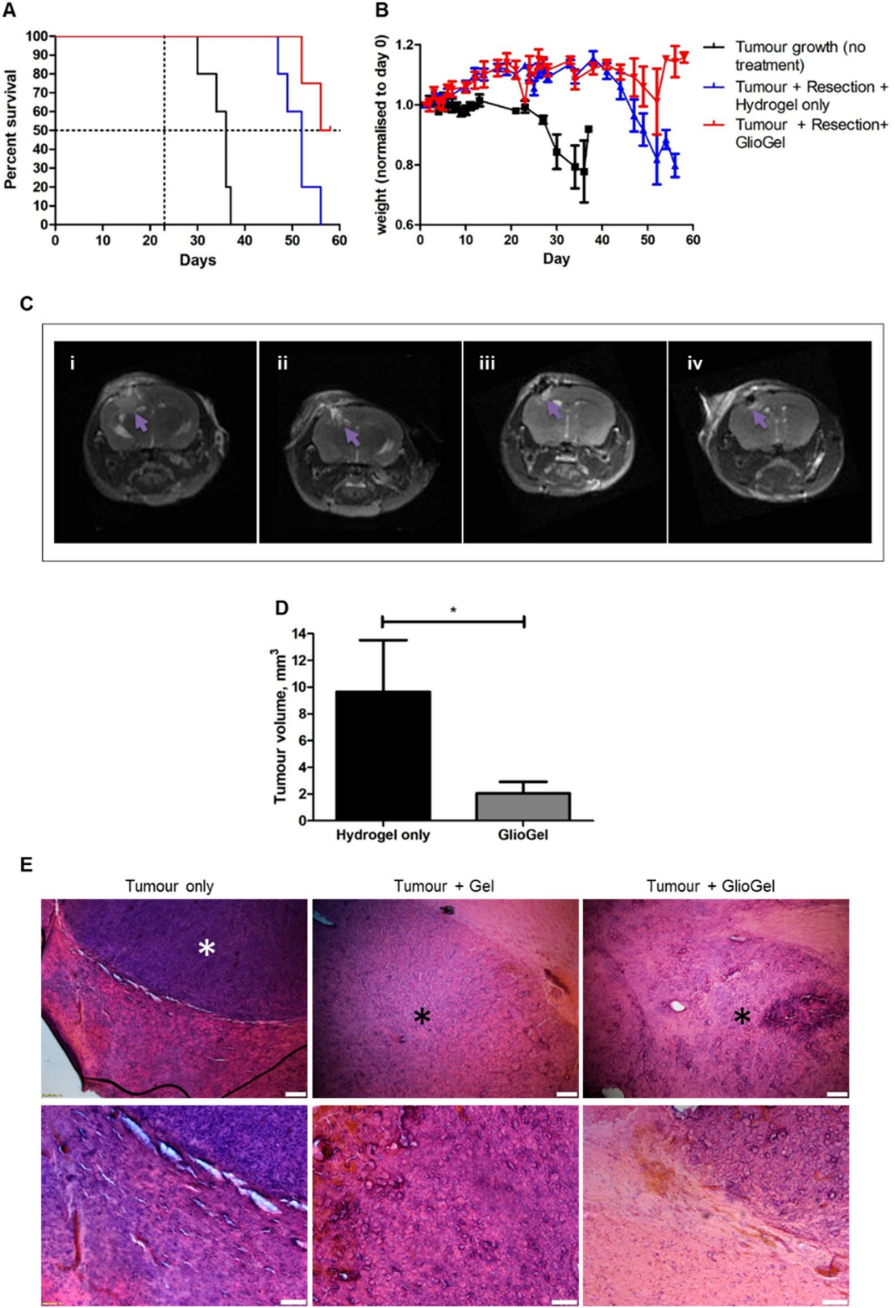
GlioGel anti-tumour effect in vivo. (**A**) Kaplan–Meier survival curves of U-87 tumour-bearing mice. Dotted lines indicate 50% survival and the day of resection surgery (day 23). Statistically significant differences were found between untreated and treatment groups: “Resection + Hydrogel only” (p = 0.0021) and “Resection + GlioGel” (p = 0.0098). (**B**) Mice weight change over time. From day 47 there are statistically significant differences between the groups “Resection + Hydrogel only” and “Resection + GlioGel” (day 47—p < 0.05; day 49 and day 54—p < 0.01; day 52 and 56—p < 0.001). (**C**) MRI of tumour regrowth one week after resection: (i) Resection + Hydrogel only. (ii) Resection + GlioGel treatment. (iii and iv) Tumour + GlioGel treatment long-term survivors. The arrow indicates the area of tumour regrowth (bright areas correspond to the tumour in T1 weighted images. In (iv), the dark area in the brain corresponds to the surgical cavity). (**D**) Tumour volume comparison one week after resection. Statistical test: non-parametric Mann–Whitney test (*p < 0.05). (Tumour growth + resection + hydrogel only, n = 5; Tumour growth + resection + GlioGel, n = 4). (**E**) Histology analysis of brain tissue. Notice haematoxylin rich tumour area which is denser in the tumour only group (control group—without resection surgery and without treatment). White * indicates dense tumour areas and black * indicates less dense tumour areas. Upper panels scale bar 200 μm; lower panels scale bar 100 μm.

Directly after surgical resection, the mice were divided into two groups, with one group receiving the CX hydrogel alone and the other group receiving the GlioGel formulation (0.6 mg/kg TMZ and 0.3 mg/kg PTX loaded into MSN-PEG). The control group, which did not undergo tumour resection (no treatment), started to show signs of distress and weight loss by day 27 after tumour cell injection, and had a median survival of 36 days (Fig. 13A,B). On the contrary, the groups that underwent resection surgery showed stable weight up to day 44. Then, weight loss was recorded for the group that received only the hydrogel, without any chemotherapy, and a median survival of 52 days was measured. The group that received the GlioGel formulation after resection had a median survival of 57 days and two long-term survivors. Statistically significant differences were found between the untreated group and the hydrogel only (p = 0.0021) and GlioGel (p = 0.0098) treatment groups. Additionally, from day 47 statistically significant differences in body weight was observed between the hydrogel only and the GlioGel groups.

The pattern of body weight change over time indicates an improvement in the welfare of the animals in the “resection + GlioGel” group, which can be explained by differences in the tumour regrowth rates. Indeed, all animals presented tumour recurrence when scanned by MRI at day 32 (Fig. 13C). However, the average tumour volume for the hydrogel only group was 9.6 mm^3^ compared to 2 mm^3^ for the GlioGel group (Fig. 13D). Moreover, in the two long-term survivors, the resection cavity was still visible and only a tumour border was identified (Fig. 13C—iii and iv). As confirmed in our model, U-87 cells are known to generate non-invasive tumours with well-defined borders in the brain of mice^58^, which are suitable to assess tumour regrowth post-resection. Alternative models to address tumour infiltration have been reported, such as invasive GBM xenografts in immunodeficient mice implanted using D-270 MG cells^59^.

Overall, our results showed that local delivery of free TMZ and PTX-loaded MSN-PEG carried by the GlioGel increased survival in GBM bearing mice without weight loss, which can be explained by the delay of tumour recurrence. Interestingly, the histological analysis in Fig. 13E shows that, after treatment, the tumoral tissue regrows with a lower density as compared to the untreated tumoral tissue (no surgery group, and resection + hydrogel only group). Given the limited effect of the standard oral TMZ treatment^59^, the GlioGel approach expands the possibility of combination treatments in an effort to address the current severe shortcomings of GBM therapy.

## Conclusion

The local delivery of chemotherapy to GBM tumours represents a powerful approach to improve treatment outcomes for this severe disease. In this paper, a new formulation (GlioGel) containing free TMZ and PTX-loaded nanoparticles was developed for the local treatment of GBM and evaluated in vitro and in a pre-clinical GBM mouse model post tumour resection. The nanoparticle component of GlioGel, PEGylated MSN, was designed to provide a triggered drug release in the reducing intracellular environment, and it showed the ability to penetrate GBM spheroids to a higher extent as compared to non-PEGylated nanoparticles. Furthermore, the addition of PTX release to the standard TMZ treatment was effective in inhibiting spheroid growth over time. The GlioGel formulation was implanted into the mice brain after resection surgery having intimate contact with the resection cavity walls. The new formulation was effective in slowing down the tumour regrowth in vivo, increasing the survival of mice bearing U-87 tumours and improving their welfare. In conclusion, GlioGel represents a viable option to be further explored in the development of novel GBM local treatments. This formulation opens the possibility to develop personalized local treatment options for GBM in the future.

## Supplementary Information

Supplementary Information.

## Data Availability

The datasets used and/or analysed during the current study are available from the corresponding author on reasonable request.

## Abbreviations

GBM: Glioblastoma multiforme
TMZ: Temozolomide
PTX: Paclitaxel
MSN: Mesoporous silica nanoparticles
