# Supplementary material for: Nanocomposite formulation for a sustained release of free drug and drug-loaded responsive nanoparticles: an approach for a local therapy of glioblastoma multiforme
Source: Sci Rep. 2023 Mar 29;13:5094. doi: 10.1038/s41598-023-32257-5 (PMC10060267; doi:10.1038/s41598-023-32257-5)
Supplement: Supplementary file 1 — Supplementary Information. [file 41598_2023_32257_MOESM1_ESM.docx]

**Supplemental figures**

**
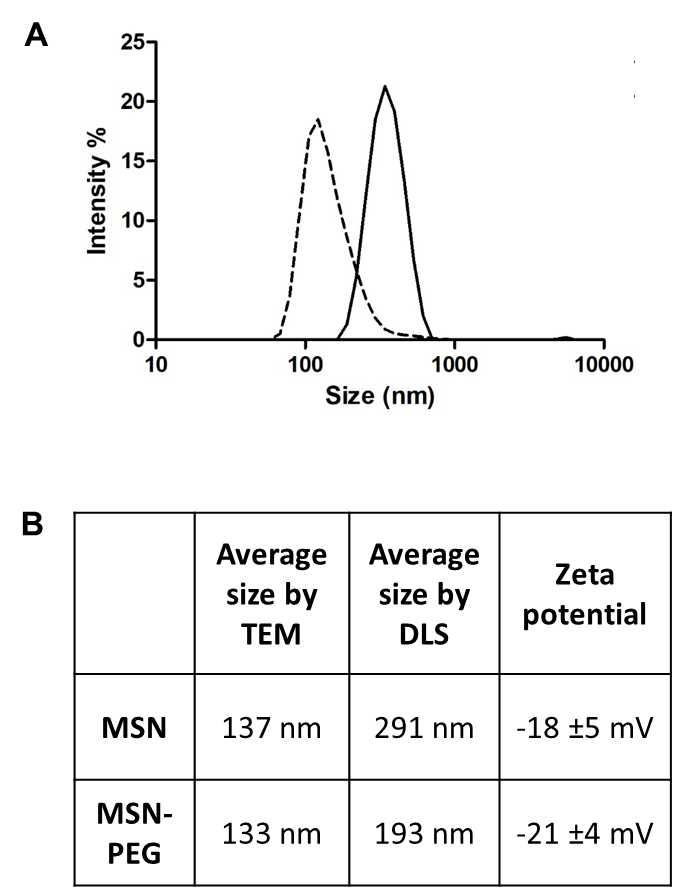
**

**Figure S1:** The morphology of the MSN did not change after functionalization with PEG. According to TEM, the average size change slightly after PEG addition. A bigger variation is measured by DLS, due to the lower number of particle aggregates after functionalization. Size distribution for MSN and MSN-PEG by DLS (**A).** Table with average size measured by DLS and by TEM, and Z-potential values (**B**).


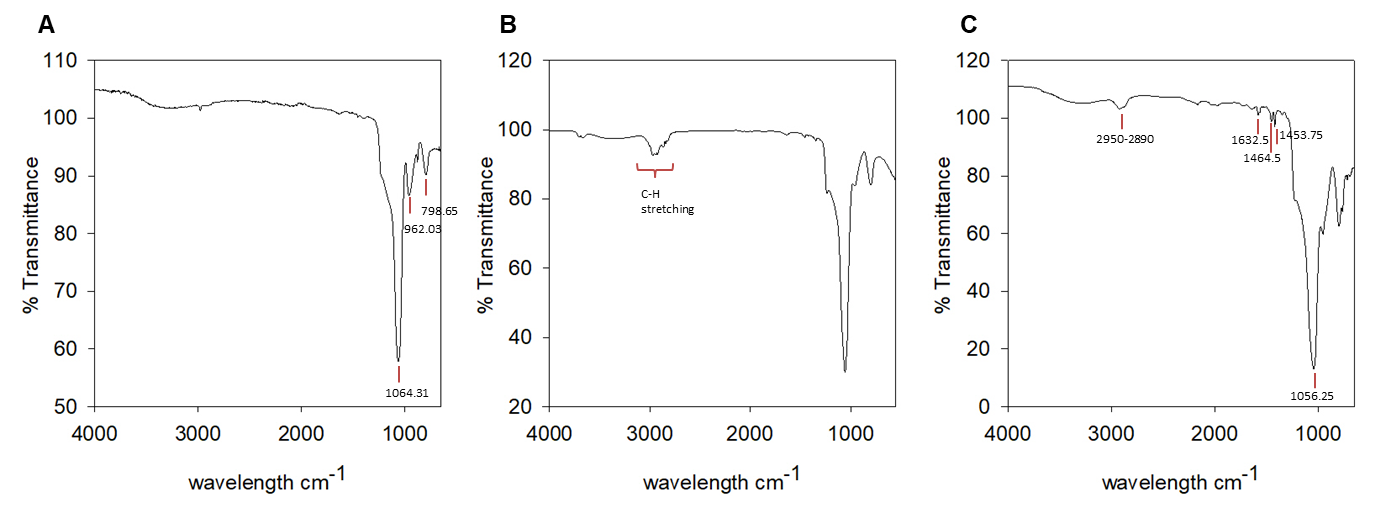


**Figure S2:** The presence of PEG on the MSN was evaluated by FTIR. **A-** FTIR spectrum of MSN shows bands correlated with Si-O-Si stretching (1064 cm^-1^) and Si-OH stretching (962, 798 cm^-1^). **B-** FTIR spectrum of MSN-PEG shows new bands correlated with C-H stretching (2840-2970 cm^-1^). **C-** FTIR spectrum of MSN-PTX-PEG shows bands correlated with C-H stretching (2950 - 2890 cm^-1^) and N-H stretching (1632, 1464,1453 cm^-1^).


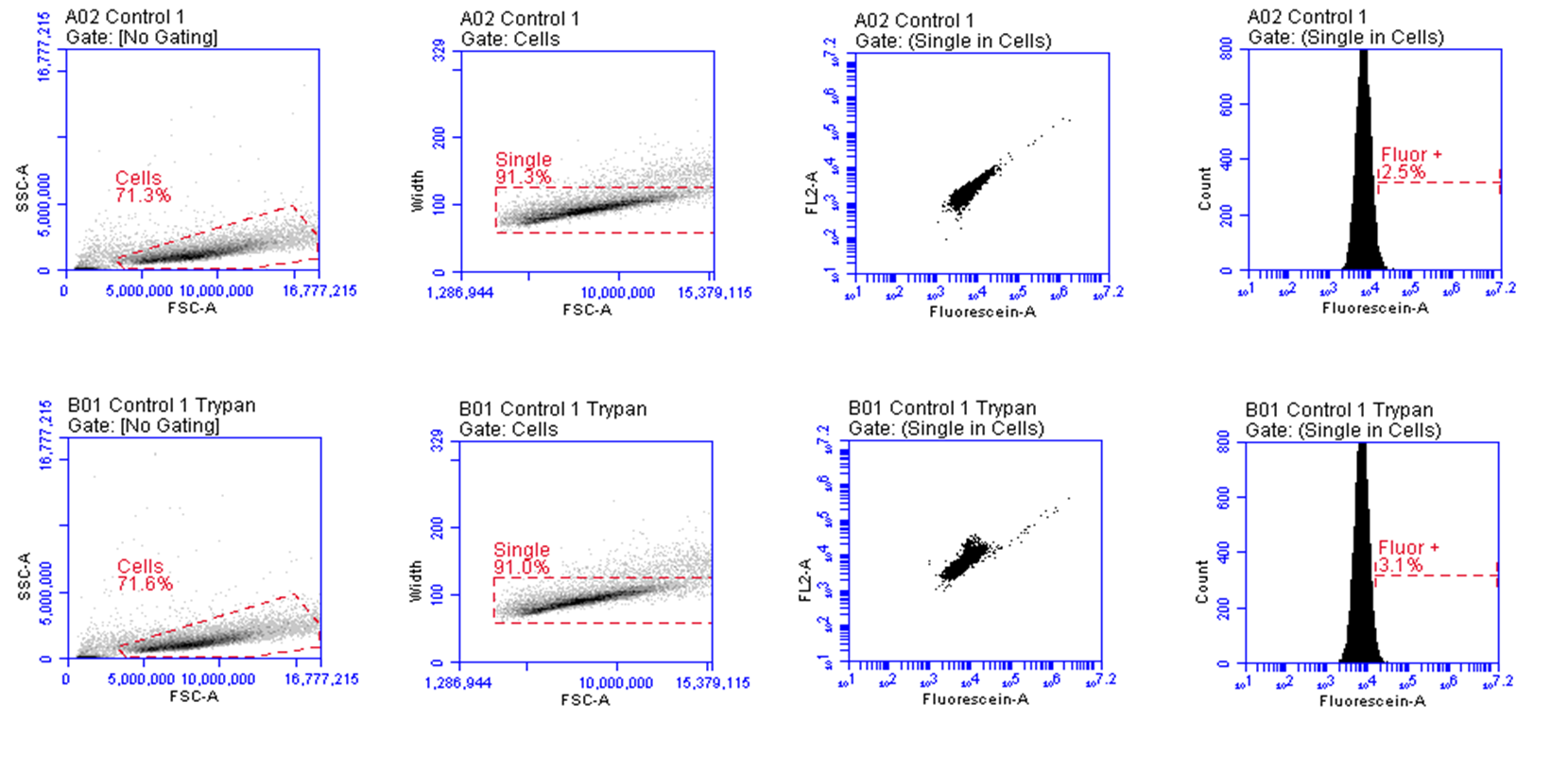


**Figure S3a:** Representative graphs from the Flow cytometer analysis of control cells before and after Trypan blue addition (Replicate 1).


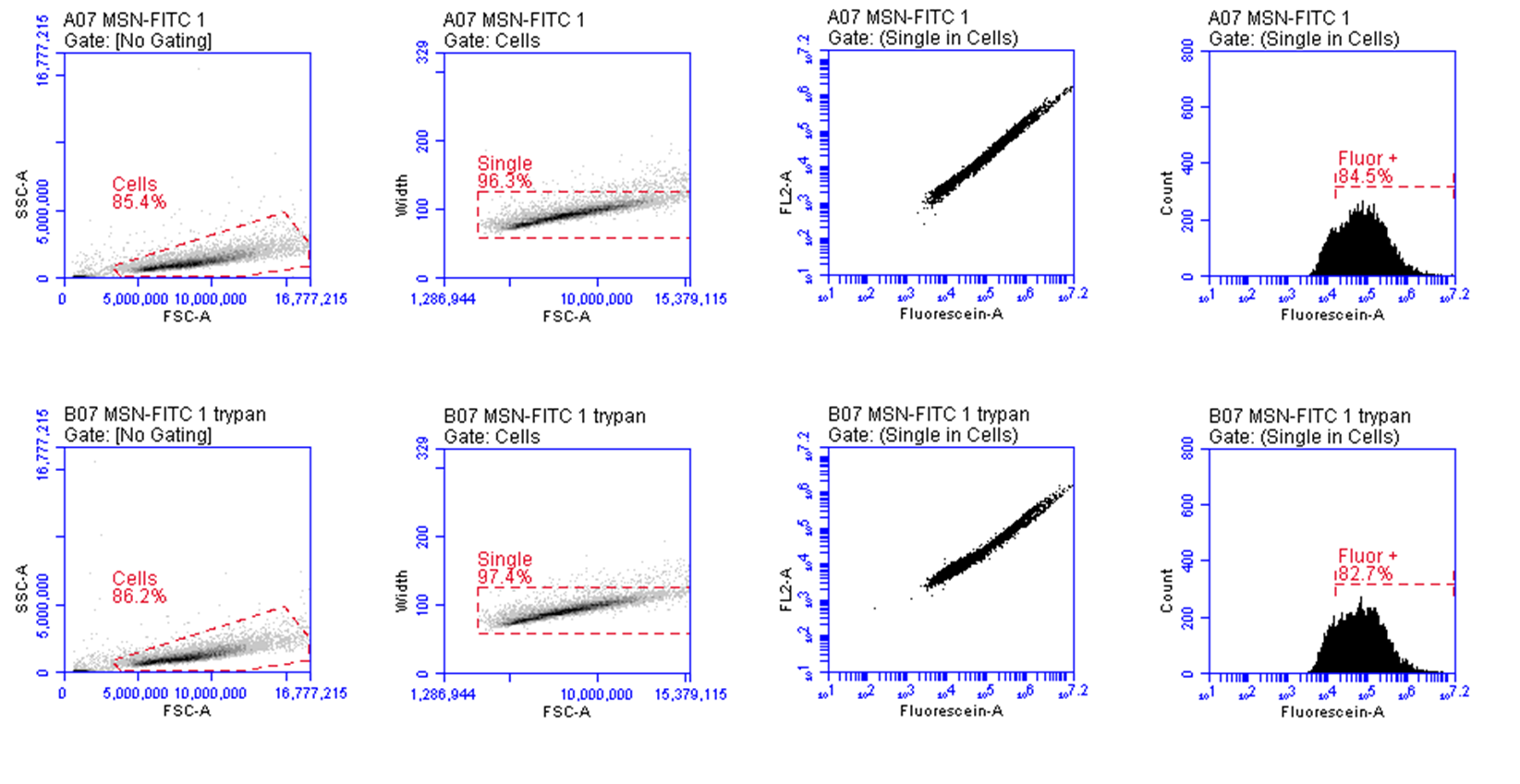


**Figure S3b:** Representative graphs from the Flow cytometer analysis of MSN-FITC treated cells before and after Trypan blue addition (Replicate 1).


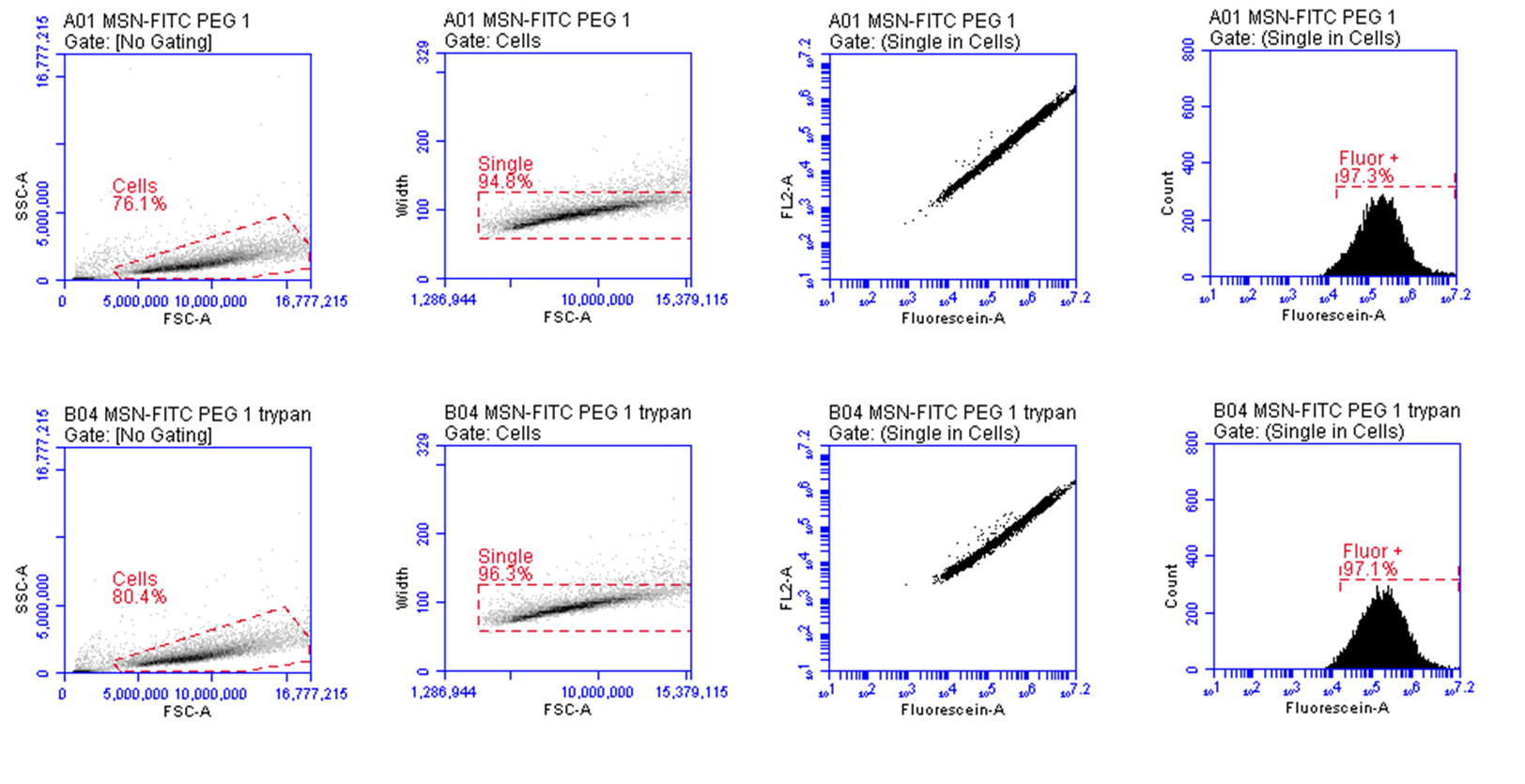


**Figure S3c:** Representative graphs from the Flow cytometer analysis of MSN-FITC-PEG treated cells before and after Trypan blue addition (Replicate 1).


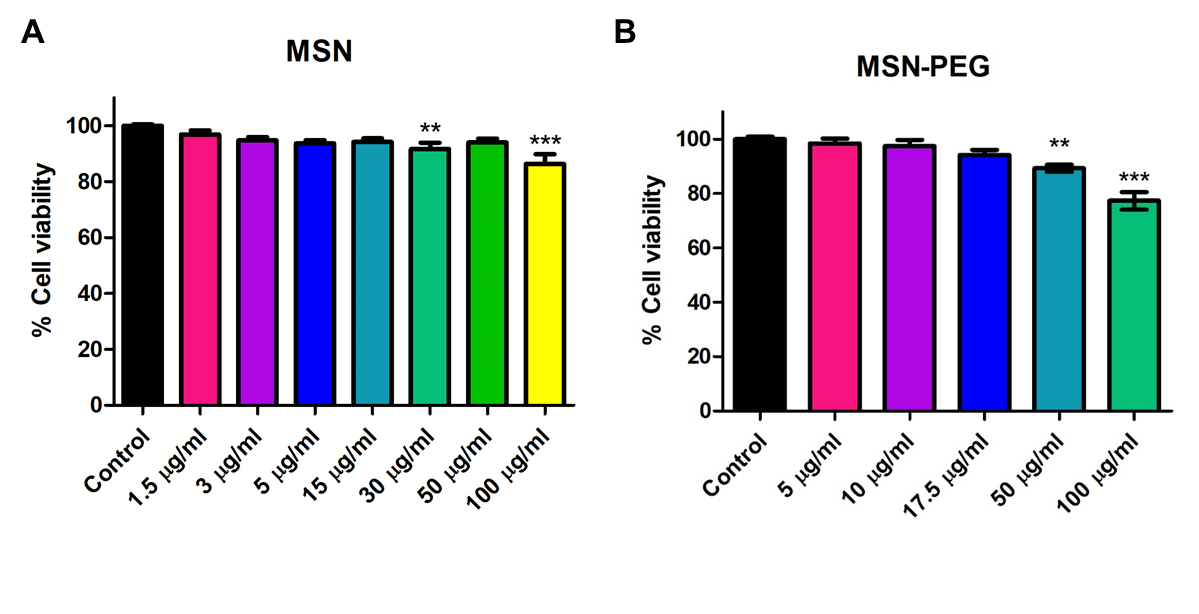


**Figure S4:** U-87 cell viability assay upon treatment with **A-** MSN and **B-** MSN-PEG, for 72 h (mean +/- SEM). Statistical test: One-way ANOVA with Dunnett’s post-test comparing all treatments versus the control. P <0.05 (*), P <0.01 (**) or P <0.001 (***).
